# The fruit morphometric variation and fruit type evolution of the stone oaks (Fagaceae, *Lithocarpus*)

**DOI:** 10.1186/s12870-023-04237-4

**Published:** 2023-04-29

**Authors:** Xi Chen, Yuanyi Qin, Dongrui Jia

**Affiliations:** 1 School of Ecology and Environmental Science, Yunnan University, Kunming, 650091 Yunnan China; 2 Yunnan Key Laboratory of Plant Reproductive Adaptation and Evolutionary Ecology and Institute of Biodiversity, Yunnan University, Kunming, 650504 Yunnan China

**Keywords:** Morphology, Trade-off, Mechanical defense, Phylogeny, Predation selection

## Abstract

**Background:**

The great species diversity of *Lithocarpus* is associated with interspecific fruit morphological variation, represented by acorn (AC) and enclosed receptacle (ER) fruit types. Species representing both fruit types co-occur in the same forests and share two distribution centers in southern China and southeastern Asia. The predation selection hypothesis suggests that the fruit morphological mechanical trade-off between two fruit types could represent divergent dispersal strategies under varied predation pressures. By integrating phylogenetic construction with fruit morphometric study, we tried to verify the predation selection hypothesis and elucidate the fruit type evolution of *Lithocarpus*, which is critical in interpreting the distribution and diversification of the genus.

**Results:**

We identified the functional trade-off between the two fruit types: ER species have bigger seeds which are enclosed mainly by receptacle representing stronger physical defense; whereas the seeds of AC species are smaller and enclosed mainly by thin pericarp representing lower mechanical protection. Despite some reversals from ER back to AC, the ancestral state reconstruction in combination with thermal analysis supports the hypothesis that ER is the derived fruit type from AC-like ancestors independently across all clades.

**Conclusion:**

Our results support the predation selection hypothesis by verifying the mechanical trade-off between the two fruit types. We propose a divergent selection theory for the two fruit types: the seed size and mechanical defense of AC species become smaller, whereas those of ER species become larger and require more morphological modifications in the receptacle. This signified the importance of the receptacle in differentiating the two fruit types and in the fruit morphological modification through evolutionary time. We found that the ER-type species evolved independently in all clades and across varied climates from tropical to warm temperate regions. As ER fruits are products of convergent evolution, we propose to examine the predation and dispersal variation between two fruit types in the future to verify whether predation selection is the reason behind fruit type evolution of the stone oaks.

**Supplementary Information:**

The online version contains supplementary material available at 10.1186/s12870-023-04237-4.

## Background

Members of Fagales, such as Fagaceae, Betulaceae and Juglandaceae, representing some of the most ecologically important trees in temperate [1] and neotropical forests [2], produce edible nuts with dry husks around. The evolution of nuts was assumed to be an adaptation to light competition, where the huge reserves of nutrients in cotyledon or endosperm confer an advantage by developing large leaves or extensive root system before they become independent [3, 4]. Meanwhile, with 10–1000 times greater caloric value than most wind-dispersed species [5], nuts became nutritious food sources for animals. Two relationships were formed between nuts and their predators: a mutualistic relationship by providing nutritional reward for the service of seed dispersal by a vertebrate [6]; and antagonism with insects as the most important pests causing pre-dispersal seed predation [5]. The nut characteristics, i.e., morphology, seed chemistry, and physical defense, interact with each other and influence seed predation and dispersal. Meanwhile, the behavior and choices of insects and vertebrates in turn significantly impact the evolution of nut characteristics [5, 7–12]. Therefore, the fruit-animal interaction is crucial in the evolution of Fagales nuts.

*Lithocarpus* Blume under Fagaceae serves as an interesting study model. Over 320 noted *Lithocarpus* species with great fruit morphological variation [13] are widely distributed from far eastern India to southern China, throughout Indochina, southern Japan, and extends through the Malayan Archipelago to Papua New Guinea [14, 15]. Their nuts are important food sources for invertebrate predators such as weevils, moths, and wasps [16, 17] as well as dispersers such as scatter-hoarding rodents [18–20] in the tropical and subtropical forests [17] of these regions, indicating that a long term fruit-animal coevolutionary relationship influences the diversification of *Lithocarpus*.

**Fig. 1:**
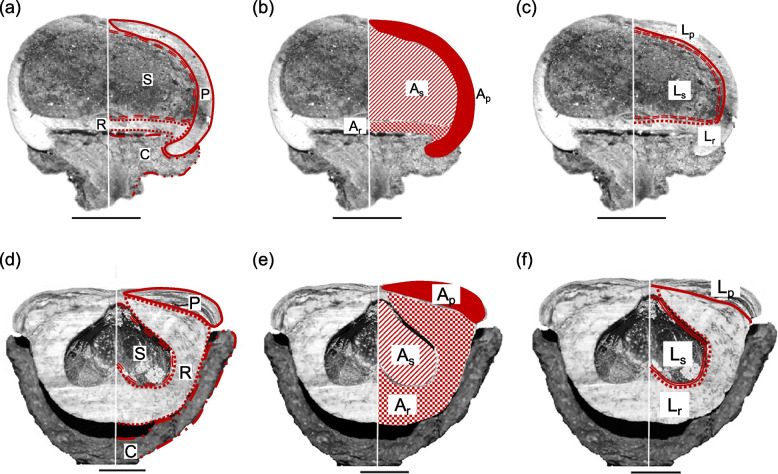
The structure of two fruit types and collected morphometric parameters on the longitudinal sectioned fruit. **a-c** and **d** to **f** represent AC and ER fruit respectively. The black scale bar and the white line in each image represent 1 cm and the longitudinal axis respectively. The original structure is displayed on the left, and the diagrammatic representation is on the right of the longitudinal axis. In **a** & **d**, the structures of cupule (C), receptacle (R), pericarp (P) and seed (S) were depicted on the left side of the fruit longitudinal section. In **b** & **e**, A_p_, A_r_, and A_s_ are the area of pericarp, receptacle and seed respectively, which were parameters for estimating the volume of pericarp (V_p_), receptacle (V_r_) and seed space (V_s_). In **c** & **f**, L_s_, L_p_, and L_r_ are length of seed space, the internal lengths of pericarp and receptacle respectively, which were collected for estimating the coverage to seed by pericarp (S_p_) and receptacle (S_r_) and seed surface area (S_s_)

The nut of *Lithocarpus* is a seed surrounded by the husk (composed of pericarp and receptacle) [17], which is further subtended by a cup or disk shaped cupule (a modified sterile branch) (Fig. 1a & d) [21, 22]. The marked interspecific variation in fruit and cupule structures has been recognized as important diagnostic characteristics of stone oaks [13–15, 23]. Cannon and Manos [24] recognized two fruit types embodying the interspecific variation of 21 tropical *Lithocarpus* species, the acorn (AC) and the enclosed receptacle (ER) fruit types (Fig. 1). Based on visual examination, the two fruit types are morphologically distinct from each other: morphologically similar to the *Quercus* acorns, AC fruits are smaller-seeded and mainly enclosed by thin pericarp, with receptacle present at the base; ER fruits are unique to the genus, larger-seeded and mainly enclosed by extended, thickened, and lignified receptacle, whereas the pericarp is greatly reduced and set on the top [24]. Fruit development study [17] identified that the fruit morphological distinction between the two types mainly resulted from the heterochrony between the receptacle and pericarp in the later fruit developmental process [17]. A potential physical–chemical trade-off between the two fruit types was proposed by examining the seed chemistry of six stone oak species [25]: AC-type species has a high concentration of antifeedants (fibers) in the seeds as chemical defense; whereas the thickened and lignified receptacle of ER-type fruits represents effective mechanical protection for the more nutritious seed. Later, it was identified that these two fruit types could represent the interspecific morphological variation of *Lithocarpus* on the genus level [26]. And even though AC-type species are more ubiquitous than ER-type species (roughly 3/1 ratio), they share two distribution centers (southern China and Southeast Asia) and often co-occur in the same forests [26, 27]. Examining the evolutionary background of these two fruit types is critical in understanding the diversification and distribution of stone oaks.

The predation selection hypothesis was proposed to elucidate the fruit type evolution of *Lithocarpus*. Seed predation and dispersal [28] strongly impact plant regeneration, distribution, and evolution [5]. The predation selection hypothesis suggests that the two fruit types represent different dispersal strategies, and their morphological distinctions are results of varied predation pressures throughout evolutionary history [25]. Smaller-sized AC fruits could attract a wider range of dispersers, but their weaker physical defense could cause higher pre-dispersal predation [26]. In contrast, ER fruits with thicker husk could inhibit insect infestation more effectively, but they can only be dispersed by larger-sized vertebrates due to their larger fruit size.

However, two aspects make it challenging to test the predation selection hypothesis. First, the limited information on insect predators and vertebrate dispersers of *Lithocarpus* fruits [26] impedes testifying this hypothesis. Second, the distinctions in seed size, fruit morphology, and mechanical defense between the two fruit types were descriptive with limited sample sizes [24, 25], as measuring the mechanical defense has been challenging. For instance, measuring the thickness of the fruit husk by calipers would include fruit positioning errors, and ignore the density variation between the receptacle and pericarp. Applying digital force gauge (SHSIWI Model SJX-200–500) to crack the husk also generates results with big variation due to fruit positioning and fruit rotating during measurement (unpublished data by Xi Chen). Applying morphometric analysis to estimate the size of varied fruit parts is a new approach to measuring the mechanical defense, which offers reliable volumetric estimates and could avoid the problems caused by direct measurements [26]. So performing the morphometric analysis with adequate sample size is necessary for clarifying the morphometric distinction and the mechanical trade-off between the two fruit types, which is crucial for verifying the predation selection hypothesis.

The unresolved phylogeny of *Lithocarpus* is another impediment in understanding the fruit type evolution of *Lithocarpus*. Interfertility, frequent interspecific gene flow, large effective population size, and so on, [29] are common difficulties in phylogenetic analysis of Fagaceae. The phylogenetic construction with 21 Bornean species [24] is the pioneer study to examine the fruit type evolution of *Lithocarpus*, which suggests ER species could have speciated from AC species in two independent linages. But the relatively low species number and the omission of subtropical species were major limitations of this study. Recently, the sequence analysis of chloroplast DNA (*atp*B-*rbc*L) and nrITS of 64 species by Yang et al. [30] provides a more complete phylogenetic relationship of *Lithocarpus*. Nevertheless, their phylogeny has limited support and the fruit type differentiation along evolutionary history remains unexamined. So it is essential to reconstruct a better-resolved phylogeny of *Lithocarpus* by employing more gene fragments while including more species which cover the entire distribution range of the genus.

In this study, we integrated phylogenetic construction and fruit morphometric study with adequate sample size to elucidate the fruit type evolution of *Lithocarpus*. We first carried out a fruit morphometric study of 168 *Lithocarpus* species to describe the interspecific and intraspecific variations of various fruit parts, and tested the morphological and mechanical trade-off between the two fruit types: the small seeds of AC-type species are mainly defended by pericarp representing low physical defense; whereas the large seeds of ER-type species are protected by thickened and lignified receptacles representing strong physical defense [25]. By coupling morphometric and phylogenetic analysis, we then investigated the evolution of fruit type and fruit morphometrics on a subset with phylogeny of 72 *Lithocarpus* species based on 5 chloroplast DNA and nrITS to test the hypothesis that ER is the derived fruit type from AC-like ancestors [24]. Lastly, to estimate the centers of origin for AC- and ER-type species, we also inferred the thermal diversification center from the present-day geographical distribution of *Lithocarpus* species. Our study is crucial in clarifying the morphological and mechanical trade-off between the two fruit types, and in verifying the predation selection hypothesis, which would bring about a deeper understanding in the fruit-animal interactions and fruit type evolution of stone oaks.

## Results

### Morphometric distinctions between the two fruit types

**Fig. 2:**
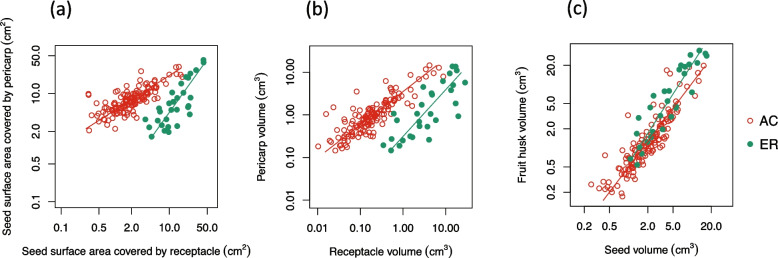
The fruit morphological variation among the 168 species and between the two fruit types. **a** the levels of seed coverage by pericarp and receptacle. **b** the volumes of pericarp and receptacle. **c** the volumes of fruit wall and seed. AC and ER stand for acorn and enclosed fruit type respectively. Each circle represents one species

The 168 study species were classified into 138 AC- and 30 ER-type species (Fig. 1, Table 1), around a 4:1 ratio. Compared to that of AC-type species, the fruit husk of ER species is generally lignified [17, 24, 25]. Here, assuming zero density difference, the fruit husk volume (V_h_) would represent total mechanical investment, and the fruit husk and seed surface area ratio (V_h_ / S_s_) would represent the relative physical protection. Based on linear mixed-effect modeling, both the seed volume (V_s_) and mechanical defense (V_h_ / S_s_) of ER species were significantly larger than those of AC species (Table 2). By performing major regression analysis to estimate the constant and allometric parameters and test the fruit type (AC or ER) dependence on them, we also identified a partition in mechanical defense between the two fruit types. The allometric exponents of AC and ER species in pericarp and receptacle coverage to the seed allometry were significantly different from each other (0.71 and 1.39, *p* = 1.48*10^–7^), and the allometric constants of which were 1.93 and 0.46, respectively (Fig. 2a). Similarly, the allometric exponents of AC and ER species in pericarp and receptacle volume allometry were also significantly different (0.88 and 0.94, *p* = 8.35*10^–3^), and the allometric constant of which were 1.77 and 0.65, respectively (Fig. 2b). These results suggest that the pericarp and receptacle provided major physical defense for the seeds of AC and ER species, respectively. Combining the results above, the stronger physical defense of ER fruits was mainly contributed by the receptacle tissue.

**Table 1:** The fruit morphometric estimations of 168 *Lithocarpus* species

Fruit type	Species	Sample	S_p_	S_r_	S_s_	V_p_	V_r_	V_s_
		number	(cm^2^)	(cm^2^)	(cm^2^)	(cm^3^)	(cm^3^)	(cm^3^)
AC	*Lithocarpus acuminatus* (Roxb.) Rehder	4	7.16	1.55	8.26	0.80	0.11	1.77
ER	*L. amygdalifolius* (Skan) Hayata	16	6.58	9.89	11.65	1.06	2.14	3.17
AC	*L. andersonii* Soepadmo	4	4.33	1.09	4.32	0.18	0.01	0.77
AC	*L. annamensis* (Hickel & A.Camus) Barnett	5	5.32	1.05	5.78	0.43	0.12	1.18
AC	*L. annamitorus* (A.Chev.) A.Camus	1	3.99	1.65	4.24	0.64	0.13	0.42
AC	*L. apoensis* (Elmer) Rehder	4	10.14	2.41	11.49	3.49	0.55	4.21
AC	*L. areca* (Hickel & A.Camus) A.Camus	60	25.87	2.99	27.43	6.20	0.39	11.11
AC	*L. auriculatus* (Hickel & A.Camus) Barnett	10	13.22	5.79	14.63	2.21	0.62	3.92
AC	*L. bacgiangensis* (Hickel & A.Camus) A.Camus	52	4.18	1.06	5.17	0.50	0.08	0.92
ER	*L. balansae* (Drake) A.Camus	1	3.34	8.78	11.08	0.32	1.47	2.41
AC	*L. bancanus* (Scheff.) Rehder	9	8.08	1.96	10.13	0.76	0.14	2.37
AC	*L. bassacensis* (Hickel & A.Camus) Barnett	5	17.86	7.74	16.90	3.90	1.51	5.56
ER	*L. beccarianus* (Benth.) A.Camus	5	2.41	9.52	9.86	0.26	4.07	2.18
AC	*L. bennettii* (Miq.) Rehder	12	6.57	1.43	7.11	0.41	0.13	1.45
AC	*L. blaoensis* (A.Camus) A.Camus	4	5.98	2.07	7.74	1.12	0.21	1.61
AC	*L. blumeanus* (Korth.) Rehder	14	6.07	1.98	7.87	0.56	0.15	1.40
AC	*L. bonnetii* (Hickel & A.Camus) A.Camus	4	2.63	0.71	2.94	0.18	0.05	0.43
AC	*L. brachystachyus* Chun	1	4.79	1.02	4.77	0.31	0.07	0.90
AC	*L. braianensis* A.Camus	4	2.22	0.99	3.06	0.24	0.05	0.40
AC	*L. brasii* Soepadmo	5	17.74	5.37	25.18	8.67	1.50	6.36
AC	*L. brevicaudatus* (Skan) Hayata	5	10.69	2.75	12.81	2.24	0.39	4.16
AC	*L. calolepis* Y.C.Hsu & H.Wei Jen	85	10.10	3.14	12.17	1.89	0.37	3.26
AC	*L. calophyllus* Chun ex C.C.Huang & Y.T.Chang	1	6.43	2.25	6.88	1.10	0.15	1.81
AC	*L. cantleyanus* (King ex Hook.f.) Rehder	3	5.39	0.64	6.13	0.31	0.04	1.17
AC	*L. carolinae* (Skan ex Dunn) Rehder	28	10.72	4.45	12.91	1.56	0.41	3.62
AC	*L. caudatifolius* (Merr.) Rehder	10	8.57	1.27	9.73	0.93	0.22	2.03
AC	*L. caudatilimbus* (Merr.) A.Camus	1	10.81	1.02	11.80	1.40	0.07	3.58
AC	*L. celebicus* (Miq.) Rehder	33	11.22	2.47	12.72	1.95	0.39	3.28
AC	*L. chrysocomus* Chun & Tsiang	1	4.03	2.20	5.80	0.32	0.17	1.14
ER	*L. cleistocarpus* (Seemen) Rehder & E.H.Wilson	11	3.16	6.08	7.12	0.27	0.54	1.61
AC	*L. clementianus* (King ex Hook.f.) A.Camus	4	8.17	3.12	9.56	1.04	0.33	2.06
AC	*L. confertus* Soepadmo	3	6.09	1.17	7.40	0.35	0.07	1.19
AC	*L. confinis* S.H.Huang ex Y.C.Hsu & H.W.Jen	7	5.75	1.35	5.66	0.45	0.10	1.13
AC	*L. conocarpus* (Oudem.) Rehder	11	4.79	0.88	5.49	0.39	0.06	1.03
AC	*L. cooperatus* (Blanco) Rehder	11	7.01	2.07	8.69	0.66	0.17	1.80
ER	*L. corneus* (Lour.) Rehder	25	10.33	18.16	17.53	3.78	13.31	6.27
AC	*L. craibianus* Barnett	38	4.40	1.23	4.77	0.37	0.09	0.90
AC	*L. crassinervius* (Blume) Rehder	2	13.86	1.62	12.69	3.53	0.08	4.36
AC	*L. cryptocarpus* A.Camus	2	20.23	17.46	18.38	7.94	8.75	3.98
AC	*L. curtisii* (King ex Hook.f.) A.Camus	7	7.19	2.20	8.75	0.88	0.21	1.76
ER	*L. cyclophorus* (Endl.) A.Camus	4	23.47	24.41	32.80	9.40	12.40	12.04
ER	*L. damiaoshanicus* C.C.Huang & Y.T.Chang	1	2.37	4.23	5.24	0.19	0.35	1.36
AC	*L. dasystachyus* (Miq.) Rehder	3	3.96	0.36	4.15	0.23	0.02	0.67
AC	*L. dealbatus* (Hook.f. & Thomson ex Miq.) Rehder	216	3.54	2.47	5.47	0.31	0.20	1.10
AC	*L. dealbatus subsp. leucostachyus* (A.Camus) A.Camus	4	4.45	1.68	5.54	0.38	0.20	1.12
AC	*L. dinhensis* (Hickel & A.Camus) A.Camus	5	3.58	1.49	4.28	0.32	0.06	0.78
ER	*L. echinifer* (Merr.) A.Camus	9	8.26	13.91	18.04	1.73	2.73	5.57
AC	*L. echinophorus* (Hickel & A.Camus) A.Camus	11	10.07	3.98	12.14	1.58	0.46	3.07
AC	*L. echinotholus* (Hu) H.Y.Chun & Huang ex Y.C.Hsu & H.W.Jen	5	8.37	2.49	10.06	0.79	0.12	2.42
AC	*L. edulis* (Makino) Nakai	8	7.06	1.83	7.64	0.60	0.15	1.83
AC	*L. eichleri* (Wenz.) A.Camus	3	9.07	2.30	10.98	1.33	0.19	2.27
AC	*L. elegans* (Blume) Hatus. ex Soepadmo	90	9.89	3.93	11.89	2.00	0.49	3.19
AC	*L. elizabethiae* (Tutcher) Rehder	4	6.84	2.33	8.72	0.91	0.13	1.77
AC	*L. elmerrillii* Chun	4	5.33	0.99	6.43	0.72	0.07	1.26
AC	*L. encleisocarpus* (Korth.) A.Camus	25	12.36	4.13	14.83	2.23	0.64	4.77
AC	*L. ewyckii* (Korth.) Rehder	12	7.79	1.57	8.80	0.70	0.19	2.12
AC	*L. falconeri* (Kurz) Rehder	7	9.56	1.71	10.46	1.42	0.16	2.68
AC	*L. farinulentus* (Hance) A.Camus	4	3.26	0.63	3.85	0.16	0.04	0.55
AC	*L. fenestratus* (Roxb.) Rehder	153	4.43	0.93	5.27	0.40	0.06	0.98
ER	*L. fenzelianus* A.Camus	1	2.59	7.24	7.27	0.19	0.83	1.49
AC	*L. ferrugineus* Soepadmo	17	5.49	1.16	6.27	0.35	0.14	1.18
AC	*L. fohaiensis* (Hu) A.Camus	6	7.30	2.88	8.81	0.99	0.23	2.09
ER	*L. fordianus* (Hemsl.) Chun	3	4.63	6.72	6.19	0.54	2.43	1.43
AC	*L. formosanus* (Skan) Hayata	2	7.49	1.54	6.86	1.04	0.15	1.51
AC	*L. garrettianus* (Craib) A.Camus	1	3.34	0.61	3.21	0.25	0.04	0.49
AC	*L. gigantophyllus* (Hickel & A.Camus) A.Camus	1	7.66	3.37	8.26	1.05	0.52	1.63
AC	*L. glaber* (Thunb.) Nakai	31	6.45	0.94	6.79	0.56	0.08	1.45
AC	*L. glutinosus* (Blume) Soepadmo	7	9.08	3.43	12.20	2.24	0.60	2.92
AC	*L. gracilis* (Korth.) Soepadmo	22	7.41	2.20	9.32	0.69	0.15	2.27
AC	*L. grandifolius* (D.Don) S.N.Biswas	53	7.43	3.15	9.40	1.21	0.26	2.27
AC	*L. hancei* (Benth.) Rehder	242	8.25	2.00	8.96	0.88	0.15	2.27
AC	*L. handelianus* A.Camus	12	9.18	3.06	12.01	1.37	0.30	2.82
AC	*L. harlandii* (Hance ex Walp.) Rehder	14	9.45	1.52	9.57	1.46	0.19	2.69
AC	*L. henryi* (Seemen) Rehder & E.H.Wilson	11	8.13	1.87	8.48	0.85	0.13	2.05
AC	*L. himalaicus* C.C.Huang & Y.T.Chang	4	8.26	2.24	9.03	1.06	0.20	2.25
AC	*L. howii* Chun	1	4.27	0.49	5.09	0.15	0.03	0.80
AC	*L. hypoglaucus* (Hu) C.C.Huang ex Y.C.Hsu & H.W.Jen	36	8.63	3.09	10.55	1.11	0.25	2.37
AC	*L. hystrix* (Korth.) Rehder	4	4.35	1.43	6.18	0.59	0.11	1.01
AC	*L. imperialis* (Seemen) Markgr	6	15.09	8.64	25.79	11.75	3.41	12.37
AC	*L. indutus* (Blume) Rehder	7	22.29	16.13	16.80	9.52	5.22	4.49
AC	*L. jacksonianus* A.Camus	3	3.50	0.89	3.36	0.19	0.03	0.52
AC	*L. jacobsii* Soepadmo	4	15.57	3.80	14.35	1.04	0.36	3.68
ER	*L. javensis* Blume	9	5.59	22.07	22.44	1.86	17.11	7.72
AC	*L. jordanae* (Villanueva) Rehder	3	6.62	0.45	8.04	0.78	0.04	1.80
ER	*L. kalkmanii* Julia & Soupadmo	2	5.88	22.53	22.60	4.99	14.94	6.69
AC	*L. kawakamii* (Hayata) Hayata	9	12.90	5.21	13.67	2.49	0.79	4.54
AC	*L. konishii* (Hayata) Hayata	19	7.92	5.30	4.97	1.62	1.64	0.98
AC	*L. korthalsii* (Endl.) Soepadmo	3	31.38	14.98	36.84	12.78	6.93	15.20
AC	*L. kostermansii* Soepadmo	3	14.90	3.62	17.10	3.97	0.52	5.47
AC	*L. kunstleri* (King ex. Hook.f.) A.Camus	1	3.03	0.49	2.95	0.19	0.04	0.34
ER	*L. lampadarius* (Gamble) A.Camus	1	15.32	16.54	15.05	3.21	4.67	4.32
ER	*L. laoticus* (Hickel & A.Camus) A.Camus	2	1.94	9.67	10.03	0.30	1.66	3.42
AC	*L. lappaceus* (Roxb.) Rehder	2	5.47	1.04	6.95	0.44	0.04	1.13
AC	*L. lauterbachii* (Seemen) Markgr	7	22.42	9.96	22.66	14.54	4.16	8.97
AC	*L. leiophyllus* A.Camus	3	3.18	0.75	2.97	0.20	0.05	0.46
ER	*L. lepidocarpus* (Hayata) Hayata	8	2.50	13.92	14.24	0.53	3.72	5.53
AC	*L. leptogyne* (Korth.) Soepadmo	16	4.49	1.41	5.46	0.30	0.09	0.86
AC	*L. licentii* A.Camus	8	9.55	3.96	10.71	1.77	0.55	2.30
AC	*L. lindleyanus* (Wall. ex A.DC.) A.Camus	5	4.97	0.87	4.94	0.62	0.06	0.91
AC	*L. litseifolius* (Hance) Chun	24	6.39	2.35	6.93	0.83	0.25	1.44
AC	*L. longanoides* C.C.Huang & Y.T.Chang	1	5.15	0.76	6.11	0.34	0.05	0.86
AC	*L. longipedicellatus* (Hickel & A.Camus) A.Camus	18	5.06	1.82	5.87	0.54	0.13	1.03
AC	*L. lucidus* (Roxb.) Rehder	13	8.21	6.05	11.43	1.87	1.26	2.68
AC	*L. luteus* Soepadmo	1	6.79	1.88	6.74	0.82	0.36	1.19
AC	*L. macphailii* (M.R.Hend.) Barnett	3	15.06	4.73	18.22	3.05	0.62	5.76
AC	*L. magneinii* (Hickel & A.Camus) A.Camus	43	6.18	2.17	7.62	0.75	0.16	1.70
ER	*L. maingayi* (Benth.) Rehder	3	4.11	23.81	22.73	0.93	19.66	8.96
AC	*L. mairei* (Schottky) Rehder	14	4.19	0.66	4.78	0.29	0.04	0.94
ER	*L. megacarpus* Soepadmo	6	41.86	42.60	39.78	13.73	14.41	16.92
AC	*L. megalophyllus* Rehder & E.H.Wilson	6	12.75	5.52	14.85	2.10	0.62	4.51
AC	*L. meijeri* Soepadmo	5	6.77	0.97	9.22	0.62	0.10	1.48
AC	*L. mindanaensis* (Elmer) Rehder	4	13.92	2.73	14.83	1.76	0.24	4.14
AC	*L. naiadarum* (Hance) Chun	16	6.32	1.00	7.17	0.53	0.09	1.63
AC	*L. nebularum* A.Camus	2	4.66	2.12	6.18	0.74	0.24	1.15
AC	*L. neorobinsonii* A.Camus	5	7.57	1.63	9.03	1.03	0.21	1.81
AC	*L. nieuwenhuisii* (Seemen) A.Camus	1	9.88	0.32	9.13	1.50	0.02	2.46
AC	*L. nodosus* Soepadmo	1	9.80	2.26	10.02	0.92	0.09	3.12
AC	*L. oblanceolatus* C.C.Huang & Y.T.Chang	3	8.46	2.05	10.73	1.33	0.17	2.70
AC	*L. obscurus* C.C.Huang & Y.T.Chang	4	7.02	1.62	7.37	0.38	0.16	1.69
ER	*L. pachycarpus* (Hickel & A.Camus) A.Camus	1	4.98	10.84	7.29	0.75	5.88	2.47
ER	*L. pachylepis* A.Camus	74	19.61	22.27	21.49	6.20	8.59	7.54
AC	*L. pachyphyllus* (Kurz) Rehder	53	7.03	3.77	8.80	1.27	0.36	2.26
AC	*L. pallidus* (Blume) Rehder	2	30.75	13.27	34.76	7.87	3.44	12.72
AC	*L. petelotii* A.Camus	1	10.32	3.84	10.50	1.79	0.42	3.07
ER	*L. platycarpus* (Blume) Rehder	6	20.25	20.35	25.30	4.53	2.97	9.92
AC	*L. polystachyus* (Wall. ex A.DC.) Rehder	113	5.89	1.52	6.99	0.64	0.12	1.42
AC	*L. pseudokunstleri* A.Camus	2	9.44	0.32	9.55	1.05	0.02	1.87
AC	*L. pseudomoluccus* (Blume) Rehder	4	22.90	13.53	26.47	6.89	2.36	8.31
AC	*L. pseudosundaicus* (Hickel & A.Camus) A.Camus	1	6.07	1.48	7.11	0.60	0.33	1.28
AC	*L. pseudovestitus* A.Camus	34	5.54	1.44	5.88	0.58	0.10	1.16
ER	*L. pseudoxizangensis* Z.K.Zhou & H.Sun	2	3.33	3.60	6.26	1.07	0.61	1.34
ER	*L. pulcher* (King) Markgr	2	27.11	31.21	26.42	10.99	18.15	9.41
AC	*L. pusillus* Soepadmo	4	8.63	3.31	9.72	1.09	0.24	2.95
ER	*L. revolutus* Hatus. ex Soepadmo	4	37.58	42.90	42.12	13.55	17.11	16.19
AC	*L. rhabdostachyus* (Hickel & A.Camus) A.Camus	8	7.68	2.79	9.94	1.35	0.44	1.92
AC	*L. robinsonii* Rehder	4	6.85	0.65	7.28	0.69	0.04	1.67
AC	*L. rosthornii* (Schottky) Barnett	3	5.49	0.69	6.55	0.29	0.03	1.25
AC	*L. rufovillosus* (Markgr.) Rehder	18	13.37	3.97	15.56	2.40	0.66	4.97
ER	*L. ruminatus* Soepadmo	1	7.04	11.77	10.43	1.05	4.41	4.09
AC	*L. scortechinii* (King ex Hook.f.) A.Camus	2	9.64	1.41	11.77	1.27	0.10	3.02
AC	*L. sericobalanos* E.F.Warb	4	10.59	5.48	13.52	2.73	1.89	3.33
AC	*L. shinsuiensis* Hayata & Kaneh	4	9.17	2.73	10.65	1.03	0.18	2.59
AC	*L. siamensis* A.Camus	8	11.18	1.10	12.71	1.01	0.08	3.39
AC	*L. silvicolarum* (Hance) Chun	25	10.43	3.91	13.22	2.10	0.39	3.62
AC	*L. skanianus* (Dunn) Rehder	2	6.94	1.59	8.24	0.85	0.14	1.45
AC	*L. sogerensis* (S.Moore) Markgr. ex A.Camus	8	9.93	2.12	11.09	4.32	0.51	2.47
AC	*L. solerianus* (Vidal) Rehder	4	8.84	1.64	10.77	0.96	0.15	2.61
AC	*L. sootepensis* (Craib) A.Camus	3	3.87	0.46	4.32	0.44	0.03	0.73
AC	*L. sphaerocarpus* (Hickel & A.Camus) A.Camus	34	4.95	0.89	5.88	0.49	0.06	1.17
AC	*L. stenopus* (Hickel & A.Camus) A.Camus	6	5.06	1.82	5.56	0.57	0.18	0.95
AC	*L. submonticolus* (Elmer) Rehder	4	5.39	1.23	7.07	0.78	0.15	1.24
AC	*L. sulitii* Soepadmo	1	10.82	1.64	13.59	1.37	0.16	3.90
AC	*L. sundaicus* (Blume) Rehder	15	8.51	2.13	10.71	1.07	0.25	2.55
AC	*L. taitoensis* (Hayata) Hayata	7	6.37	1.74	6.80	0.66	0.13	1.38
ER	*L. tenuilimbus* H.T.Chang	10	2.05	8.13	8.09	0.25	0.99	1.98
AC	*L. thomsonii* (Miq.) Rehder	5	5.32	1.31	5.04	0.77	0.15	0.80
AC	*L. touranensis* (Hickel & A.Camus) A.Camus	3	7.47	2.60	9.64	1.19	0.18	1.94
AC	*L. trachycarpus* (Hickel & A.Camus) A.Camus	7	4.55	0.60	5.41	0.31	0.05	0.99
ER	*L. truncatus* (King ex Hook.f.) Rheder	60	1.59	4.70	5.25	0.15	0.52	1.07
AC	*L. tubulosus* (Hickel & A.Camus) A.Camus	1	9.53	1.68	10.73	0.96	0.23	2.11
ER	*L. turbinatus* (Stapf) Forman	3	8.38	24.64	22.24	5.74	28.40	12.94
AC	*L. urceolaris* (Jack) Merr	11	17.68	2.87	17.53	4.32	0.69	6.00
ER	*L. uvariifolius* (Hance) Rehder	5	10.46	11.24	12.13	2.98	4.81	3.67
ER	*L. variolosus* (Franch.) Chun	27	5.31	6.45	9.17	0.75	0.72	2.24
AC	*L. vestitus* (Hickel & A.Camus) A.Camus	4	4.35	1.41	4.21	0.38	0.10	0.70
AC	*L. vinkii* Soepadmo	3	2.07	0.33	2.68	0.25	0.02	0.26
AC	*L. woodii* (Hance) A.Camus	10	16.82	5.30	17.97	3.18	0.81	5.96
ER	*L. xylocarpus* (Kurz) Markgr	162	5.02	13.74	14.26	0.69	2.39	4.69

S_s_, S_p_ and S_r_ are surface area of seed space, seed-enclosure level by pericarp and receptacle respectively. V_s_, V_p_ and V_r_ are volume of seed, pericarp and receptacle respectively. AC and ER stand for acorn (138 species) and enclosed receptacle (30 species) fruit type respectively

**Table 2:** The differences in seed size and mechanical defense between the two fruit types of the 168 species by linear mixed effect model

Morphometric dimensions	AC	t value	ER	t value
Fruit husk volume V_h_(cm^3^) / seed surface area (cm^2^)	0.19	2.11	1.54	6.37
Seed volume V_s_ (cm^3^)	2.57	10.53	5.72	5.42

AC and ER stand for acorn and enclosed receptacle fruit type, respectively. Assuming zero density difference, the fruit husk volume (V_h_) would represent total mechanical investment, so the fruit husk and seed surface area ratio (V_h_ / S_s_) would represent the relative physical protection

### The morphometric variation of AC and ER fruits

**Fig. 3:**
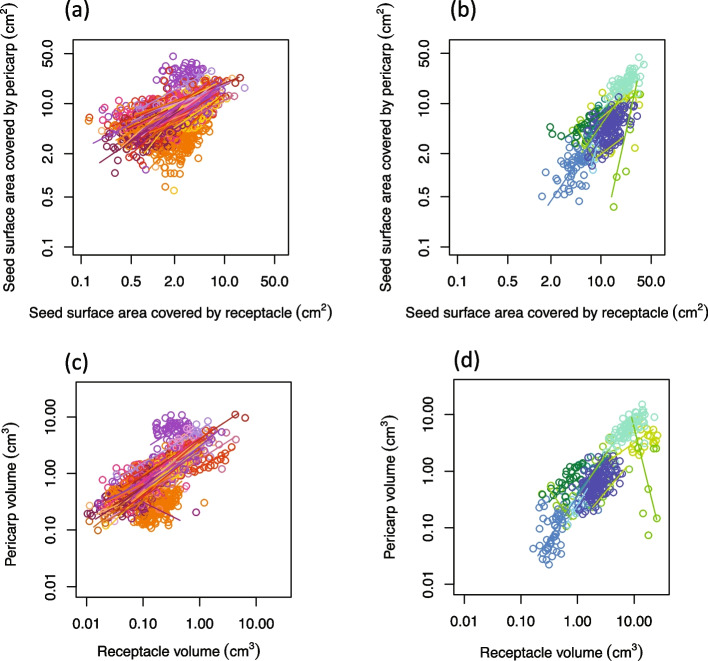
The intraspecific variations of fruit morphological dimensions within the two fruit types. **a** the seed coverage by pericarp and receptacle of the AC species. **b** the seed coverage by pericarp and receptacle of the ER species. **c** the volume of pericarp and receptacle of the AC species. **d** the volume of pericarp and receptacle of the ER species. Each circle represents a single fruit, and colors correspond to the 51 AC and 11 ER species with no less than eight fruits respectively

Representing total mechanical defense, fruit husk volume increased with species’ seed size (Fig. 2c). Receptacle volume increased accordingly with pericarp volume for most of the 168 species (Fig. 3c & d). However, two exceptions indicated negative allometric slope: *L. ferrugineus* is a small-sized AC species with extremely thin pericarp (Fig. S1 d-f), and *L. javensis* is an ER species with great interspecific variation (Fig. S1 a-c). The morphometric variation between fruit types was greater than interspecific variation within each fruit type (Fig. 2 and 3).

### Evolution of fruit types and fruit morphology

**Fig. 4:**
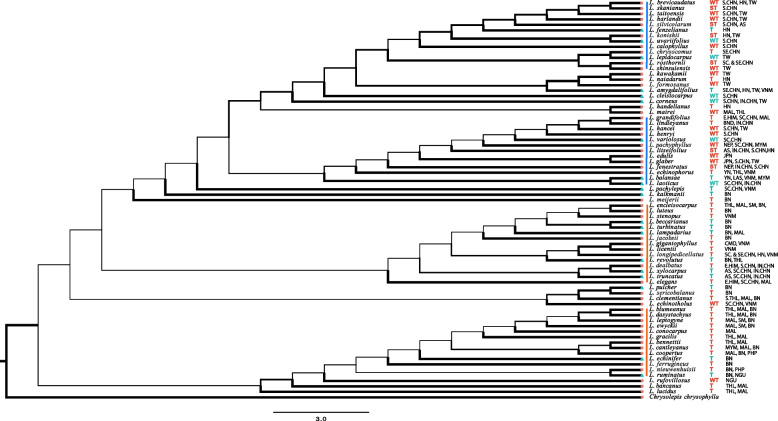
The thermal distribution center of 72 *Lithocarpus* species based on maximum likelihood phylogeny. Thicker line represents a higher support value. AC and ER type species were represented by red circles and blue triangles respectively. The thermal distribution center is represented by mean annual temperature (MAT) of the distribution median. From top to bottom, the box in pink, blue, green, purple and oranges are the main clades. T, ST and WT stands for tropical, subtropical and warm temperate regions respectively. The orange and blue lines represent two major tropical and two major warm temperate lineages respectively. The current distribution is labeled for each species after the thermal distribution center. Countries and larger regions are represented by three letters (CHN-China, THL-Thailand, LAS-Laos, VNM-Vietnam, MAL-Malaysia, BND-Bangladesh, CMD-Cambodia, MYM-Myanmar, NEP-Nepal, JPN-Japan, PHP-Philippines, NGU-New Guinea, HIM-Himalaya, IN.CHN-Indo China). Islands and lower administrative divisions are represented by two letters (HN-Hainan, TW-Taiwan, AS-Assam, YN-Yunnan, BN-Borneo, SM-Sumatra)

**Fig. 5:**
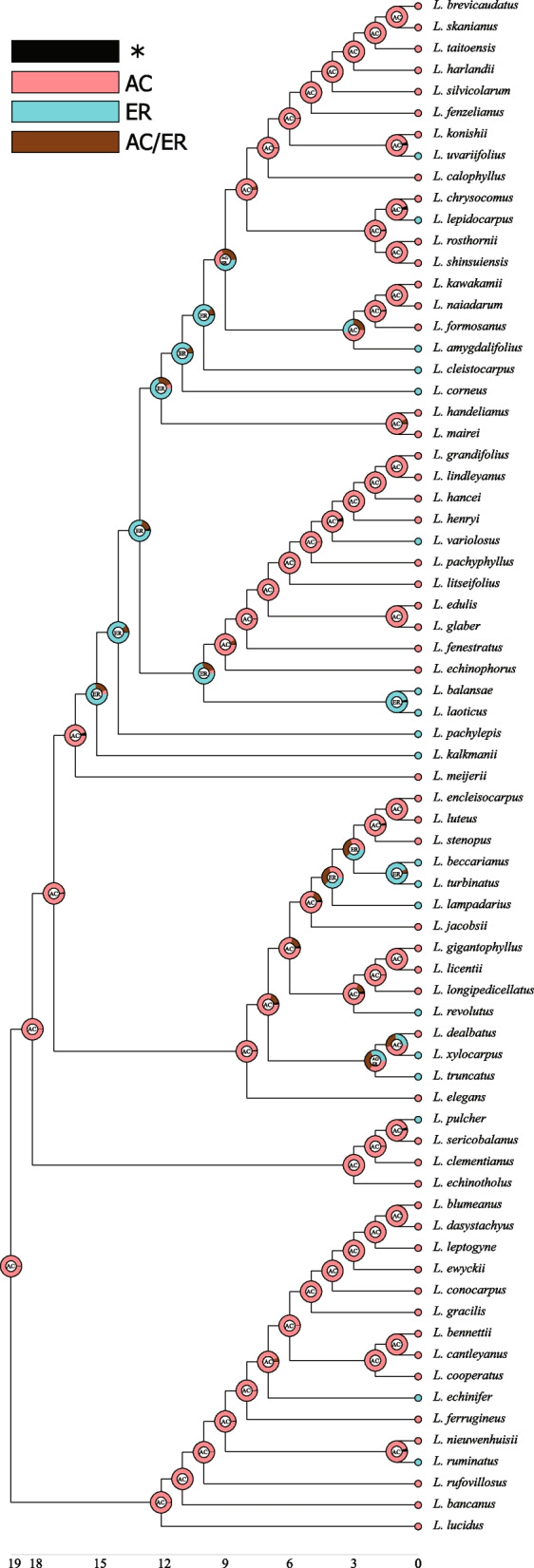
Ancestral state reconstruction of fruit type based on the ML phylogeny of 72 *Lithocarpus* species with cpDNA + nrITS. The fruit type of each species is coded by a colored dot as explained in the legend. Pie charts at nodes show the relative proportion of alternative ancestral state estimated in RASP. AC and ER represent acorn and enclosed receptacle fruit type respectively. AC/ER represents it could be either AC or ER status. The asteroid mark represents hidden probabilities

Topologies of cpDNA + nrITS (72 species), cpDNA (58 species), and nrITS (66 species) phylogenetic trees were consistent with one another (Fig. 4 & S2, Table S1 & S2). Topologies of 72 species’ phylogeny reconstructed by ML (Fig. 4) and Bayesian analyses (Fig. S3) were also largely congruent with each other. We applied the fully bifurcating ML tree to perform ancestral state reconstructions. The reconstruction showed a major transition from AC to ER fruit in the deeper branches, and after which there were several reversals to the ancestral state (AC). Besides the ER-type species that retained their fruit type, there were also ER fruits derived from acorn-like fruits in the clades independently (Figs. 4 & 5). This was further supported by the repetitive occurrence of the ER fruit type in all clades and subclades of the phylogenetic tree reported by Yang et al. (Fig. S4) [30].

**Fig. 6:**
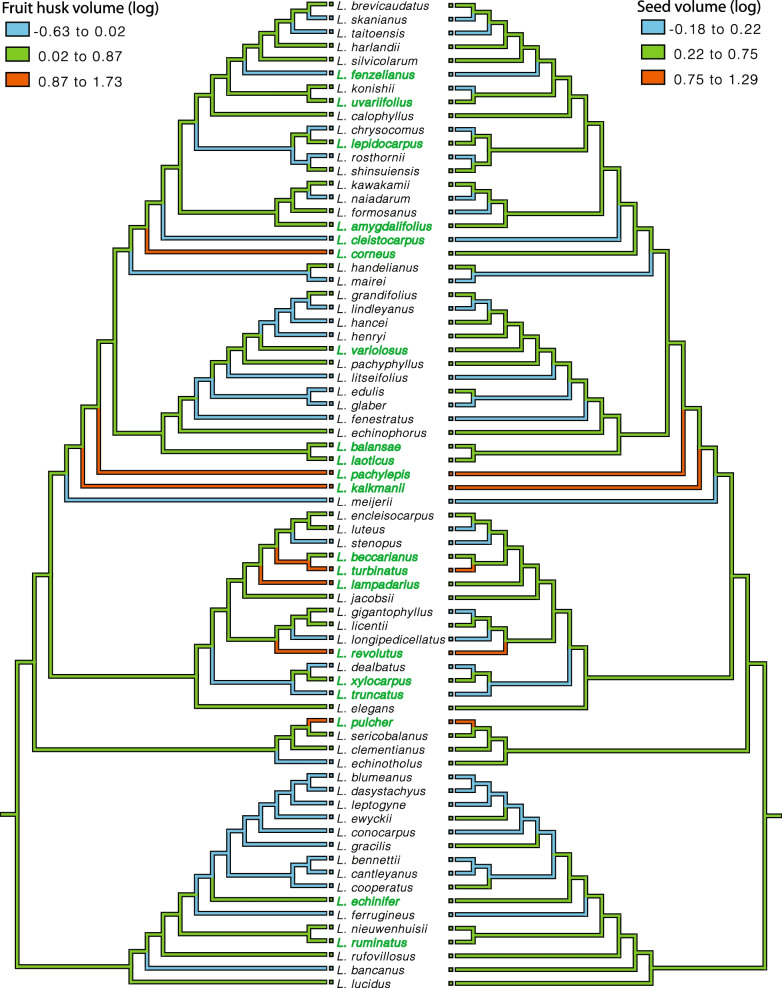
Ancestral state reconstruction of husk and seed volumes based on the ML phylogeny of the 72 *Lithocarpus* species. Estimated ancestral morphometric values are coded by colored branches as explained in the legend within the figure. The AC and ER-type species are colored by black and green respectively

**Fig. 7:**
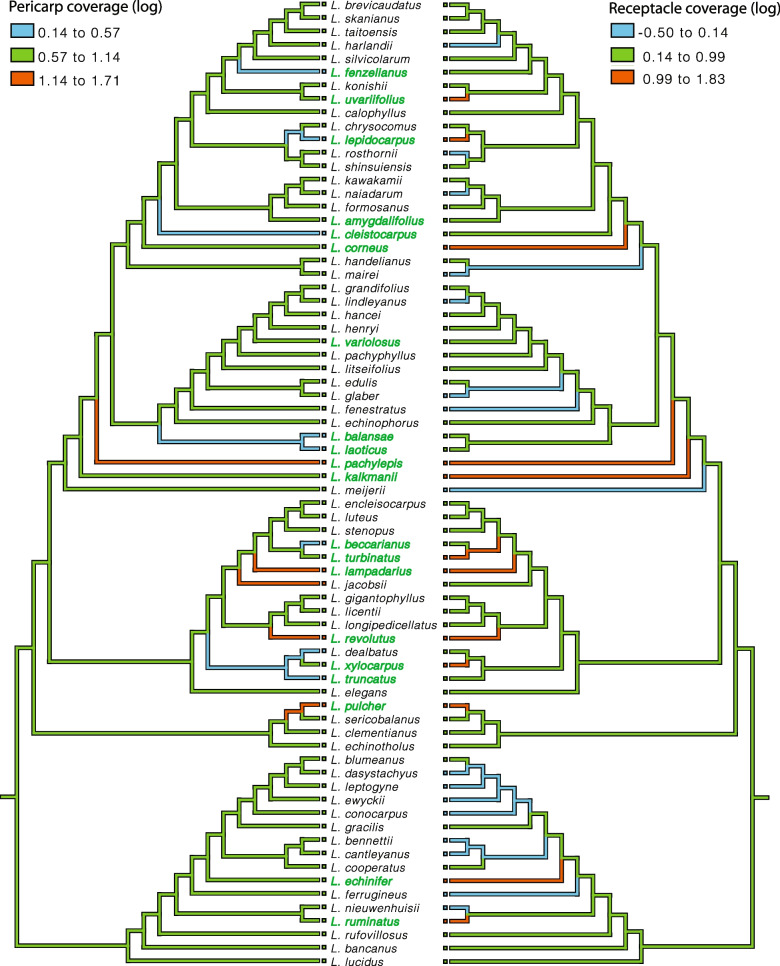
Ancestral state reconstruction of pericarp and receptacle enclosure to seed based on the ML phylogeny of the 72 *Lithocarpus* species. Estimated ancestral morphometric values are coded by colored branches as explained in the legend within the figure. The species names of AC and ER-type species are colored by black and green respectively

Fruit types showed low phylogenetic signal (K = 0.24), suggesting that ER-type species are phylogenetically distantly related to each other. The low phylogenetic signal of fruit types was further supported by the low phylogenetic signals (K < 1) of all six morphometric dimensions (S_p_, S_r_, S_s_, V_p_, V_r_ and V_s_) (Table S3). Ancestral state reconstruction of the morphometric dimensions showed that around half of the AC-type (28 out of 52) species reduced in seed size throughout the evolutionary time course, while half remained the same (Fig. 6). The increase in seed size was only observed for 5 ER-type species, while the seed size for the rest 15 species remained the same. The fruit husk volume representing physical defense changed accordingly with the seed size for both fruit types (Fig. 6). Congruent with the finding that the receptacle is the main tissue differentiating the morphology of the two fruit types (Tables 1 & 2), the reduction and increase in the receptacle coverage to the seed was observed in both AC- and ER-type species (Fig. 7). Besides *L. jacobsii*, the only exception exhibiting an increase in pericarp coverage, the pericarp coverage for the rest AC-type species remained the same. More ER-type species exhibited decrease rather than increase in pericarp coverage to the seed (8 compared to 4 species). The evolutionary changes in pericarp and receptacle volumes for both fruit types exhibited a similar pattern to that of the seed coverage by pericarp and receptacle (Fig. S5). There was an increase in both receptacle and pericarp coverage to the seed (Fig. 7) for four ER species, i.e., *L. pachylepis*, *L. lampadarius*, *L. revolutus* and *L. pulcher* (Fig. S6). These four species shared an intermediate fruit morphology between AC and ER fruits: their receptacle was thickened and extended while the pericarp was not obviously reduced. Unlike two fruit types with clear morphological distinctions, this intermediate morphology suggested that these four species were heavily protected by both tissues.

### The thermal distribution of two fruit types

Based on the distribution median of present-day mean annual temperature (MAT) (Fig. 4, Table 3 & S4), the thermal distribution centers of the 72 examined species were mostly concentrated in the tropics (45 species), followed by warm temperate forests (21 species), and subtropical forests (6 species). The tropical-centered species were mainly clustered into two major lineages. The thermal distribution centers for the other two major lineages were warm temperate forests mainly, with a few species occasionally appearing in the tropics and in the subtropics (Fig. 4). For ER-type species, the thermal distribution centers for 14 and 6 species were tropical and warm temperate regions, respectively. The thermal distribution of AC species was mostly in the tropics (31 species), with 15 species in warm temperate and 6 species in subtropical regions. Except for 13 species, the thermal distribution centers based on the median annual temperature were mostly congruent with those based on MAT (Table 3 & S4). Among the exceptions, the thermal distribution centers of 11 species based on median and mean annual temperatures were tropical and subtropical, respectively, indicating a negative skewness in distribution towards the colder region; and the thermal distribution centers of the remaining two species (*L. calophyllus* and *L. corneus*) based on median and mean annual temperatures were warm temperate and subtropical regions, respectively, indicating a positive skewness in distribution towards warmer climate.

**Table 3:** The mean and median thermal distribution center of 72 species

Fruit type	Species	Median MAT (^o^C)	Median region	Mean MAT (^o^C)	Mean region
AC	*L. bancanus*	26.2	Tropical	26.2	Tropical
AC	*L. bennettii*	26.2	Tropical	26.2	Tropical
AC	*L. blumeanus*	27.0	Tropical	26.4	Tropical
AC	*L. brevicaudatus*	17.8	Warm temperate	18.7	Warm temperate
AC	L. calophyllus**	19.0	Warm temperate	19.4	Subtropical
AC	*L. cantleyanus*	26.5	Tropical	26.3	Tropical
AC	L. chrysocomus*	22.3	Tropical	20.8	Subtropical
AC	*L. clementianus*	25.9	Tropical	26.0	Tropical
AC	*L. conocarpus*	26.1	Tropical	26.0	Tropical
AC	*L. cooperatus*	25.9	Tropical	26.0	Tropical
AC	*L. dasystachyus*	25.0	Tropical	25.0	Tropical
AC	L. dealbatus*	23.4	Tropical	21.9	Subtropical
AC	L. echinophorus*	22.2	Tropical	21.4	Subtropical
AC	*L. echinotholus*	18.6	Warm temperate	18.6	Warm temperate
AC	*L. edulis*	16.1	Warm temperate	16.1	Warm temperate
AC	L. elegans*	24.4	Tropical	21.7	Subtropical
AC	*L. encleisocarpus*	26.2	Tropical	26.1	Tropical
AC	*L. ewyckii*	25.9	Tropical	25.8	Tropical
AC	*L. fenestratus*	21.1	Subtropical	19.6	Subtropical
AC	*L. ferrugineus*	25.0	Tropical	25.0	Tropical
AC	*L. formosanus*	17.5	Warm temperate	17.5	Warm temperate
AC	*L. gigantophyllus*	25.0	Tropical	25.0	Tropical
AC	*L. glaber*	17.8	Warm temperate	18.0	Warm temperate
AC	*L. gracilis*	25.9	Tropical	25.8	Tropical
AC	L. grandifolius*	24.4	Tropical	21.7	Subtropical
AC	*L. hancei*	17.8	Warm temperate	18.4	Warm temperate
AC	*L. handelianus*	24.1	Tropical	24.1	Tropical
AC	*L. harlandii*	17.8	Warm temperate	18.4	Warm temperate
AC	*L. henryi*	16.2	Warm temperate	15.4	Warm temperate
AC	*L. jacobsii*	25.0	Tropical	25.0	Tropical
AC	*L. kawakamii*	17.5	Warm temperate	17.5	Warm temperate
AC	*L. konishii*	20.8	Subtropical	20.8	Subtropical
AC	*L. leptogyne*	25.9	Tropical	25.8	Tropical
AC	*L. licentii*	22.2	Tropical	22.2	Tropical
AC	*L. lindleyanus*	27.0	Tropical	26.0	Tropical
AC	*L. litseifolius*	21.1	Subtropical	20.4	Subtropical
AC	L. longipedicellatus*	22.3	Tropical	19.8	Subtropical
AC	*L. lucidus*	26.2	Tropical	26.2	Tropical
AC	*L. luteus*	25.0	Tropical	25.0	Tropical
AC	*L. mairei*	14.9	Warm temperate	14.9	Warm temperate
AC	*L. meijeri*	25.0	Tropical	25.0	Tropical
AC	*L. naiadarum*	24.1	Tropical	24.1	Tropical
AC	*L. nieuwenhuisii*	26.0	Tropical	26.0	Tropical
AC	*L. pachyphyllus*	18.3	Warm temperate	18.3	Warm temperate
AC	*L. rosthornii*	19.4	Subtropical	19.3	Subtropical
AC	*L. rufovillosus*	16.1	Warm temperate	16.1	Warm temperate
AC	*L. sericobalanos*	27.2	Tropical	26.6	Tropical
AC	*L. shinsuiensis*	17.5	Warm temperate	17.5	Warm temperate
AC	*L. silvicolarum*	21.1	Subtropical	20.4	Subtropical
AC	*L. skanianus*	20.1	Subtropical	19.9	Subtropical
AC	*L. stenopus*	22.2	Tropical	22.2	Tropical
AC	*L. taitoensis*	17.8	Warm temperate	18.4	Warm temperate
ER	L. amygdalifolius*	22.2	Tropical	21.2	Subtropical
ER	L. balansae*	22.1	Tropical	21.5	Subtropical
ER	*L. beccarianus*	25.0	Tropical	25.0	Tropical
ER	*L. cleistocarpus*	17.8	Warm temperate	18.3	Warm temperate
ER	L. corneus**	18.7	Warm temperate	19.7	Subtropical
ER	*L. echinifer*	25.0	Tropical	25.0	Tropical
ER	*L. fenzelianus*	24.1	Tropical	24.1	Tropical
ER	*L. kalkmanii*	25.0	Tropical	25.0	Tropical
ER	*L. lampadarius*	25.5	Tropical	25.6	Tropical
ER	*L. laoticus*	17.3	Warm temperate	16.7	Warm temperate
ER	*L. lepidocarpus*	17.5	Warm temperate	17.5	Warm temperate
ER	L. pachylepis*	22.2	Tropical	19.8	Subtropical
ER	*L. pulcher*	25.0	Tropical	25.0	Tropical
ER	*L. revolutus*	26.1	Tropical	26.1	Tropical
ER	*L. ruminatus*	25.0	Tropical	25.0	Tropical
ER	L. truncatus*	22.1	Tropical	20.4	Subtropical
ER	*L. turbitus*	25.0	Tropical	25.0	Tropical
ER	*L. uvariifolius*	17.5	Warm temperate	18.6	Warm temperate
ER	*L. variolosus*	15.7	Warm temperate	15.7	Warm temperate
ER	L. xylocarpus*	22.1	Tropical	20.4	Subtropical

MAT stands for mean annual temperature corresponding to the warm index (Kira, 1991). One aster mark (*) indicated that thermal region identified based on median MAT is hotter than that of mean MAT; two aster mark (**) indicated that thermal region identified based on mean MAT is hotter than that of median MAT

## Discussion

The marked interspecific fruit morphological variation and the two major fruit types of stone oaks represent the species diversity of the genus [17, 27]. Examining fruit morphological variation and phylogenetic relationship is important in understanding the diversification and fruit evolution of *Lithocarpus*. We studied the morphometrics of 2,613 fruit specimens of 168 species, which represent over half of the species of *Lithocarpus* and encompass a wide geographic distribution. Despite the frequent co-occurrence of the two fruit types [26, 27], AC-type species are more diverse than ER-type species (4:1 ratio, Table 1), which is consistent with previous study [27]. As the morphological variation between the fruit types was larger than within each fruit type or within each species (Figs. 2 & 3), the two fruit types are key to representing fruit morphological variation of the genus.

Instead of applying direct measurements involving various errors, the fruit morphometric analysis successfully quantified the volume of varied fruit parts and identified a major fruit morphological and mechanical trade-off between the two fruit types: smaller-seeded AC fruits were mainly enclosed within thin pericarp, representing weaker mechanical protection; larger-seeded ER fruits, on the other hand, were covered by thickened and lignified receptacle tissue, indicating stronger mechanical defense (Table 2 and Fig. 2). The mechanical trade-off between the two fruit types serves as strong evidence for the predation selection hypothesis. To further testify this hypothesis, examining the seed predation and dispersal variation between AC- and ER-type species is essential. One thing to point out is that the actual mechanical defense difference between the two fruit types should be even higher than our estimate, as the observed lignification in receptacle tissue in ER-types species was not assessed by the morphometric method. We identified that receptacle variation was the major contributor to the fruit-type morphometric distinctions, and this was more obvious for smaller fruits (Fig. 2a & b). Larger-fruited species sometimes exhibit an intermediate morphology in which the seeds were covered by both pericarp and receptacle (Fig. S4), as they require high mechanical investment to effectively defend their seeds.

The morphometric and molecular phylogeny study of 21 Bornean *Lithocarpus* species [24] suggested that the ER fruit is the derived fruit type that has occurred on at least two separate lineages for tropical stone oaks. Despite a few reversals from ER to AC, we found that ER fruits derived independently multiple times, across all clades on the phylogeny of 72 species, and across climatic ranges from tropical to warm-temperate regions (Figs. 4 & 5). Resembling the common acorn of *Quercus* in general, AC fruit represents the ancestral form of *Lithocarpus*, which has experienced less evolutionary modification in pericarp development (Fig. 7 & S5). This result supports the hypothesis that ER is the derived fruit type from the AC-like ancestors [24].

Even though we employed four additional cpDNA fragments compared to the previous study [30], some resulting topologies remained with limited support (Fig. 4). However, the repeated occurrence of ER fruits on our phylogenetic tree and previous phylogenetic trees [24, 30] suggests the convergent evolution of ER fruits on different lineages across different regions (Fig. 4 & S4). In addition, the low phylogenetic signal of the two fruit types suggests that ER-type species are distantly related to each other, which further supports the convergent evolution of ER-type species.

As ER-type species are signified by their thickened and lignified fruit husk, Cannon and Manos [24] suggested that the ER fruit was a result of strong directional selection toward mechanical protection against seed predators. Based on the two-fruit-type morphometric distinction (Table 2, Figs. 2 & 3) and ancestral state reconstruction of six fruit morphometrics (Figs. 6, 7 & S5), we propose to modify the directional selection theory to divergent selection of the two fruit types. In their evolutionary history, AC- type species tended to reduce their seed size, mechanical defense and receptacle coverage, with little change in pericarp; whereas ER-type species tended to increase in seed size, mechanical defense, with major modification in receptacle. Compared to the little variation for AC-type species, the evolutionary change of pericarp varied greatly among ER-type species (Fig. 7 & S5). This indicates that more modifications in receptacle development are required to achieve the fruit husk change (especially for ER-type species), which signifies the important role of pericarp in the evolution of fruit morphology.

The biogeographical study of 91 Chinese stone oaks signifies the impact of mean annual temperature on the fruit morphometric variation [27], so inferring the thermal diversification center based on current geographical distribution is critical in understanding the diversification and fruit-type differentiation of *Lithocarpus*. The thermal distribution pattern of 72 species (Fig. 4) suggests that the phylogenetic radiation of species-rich *Lithocarpus* involved two geographic speciation centers: tropical versus warm temperate regions. The AC-type species are more diverse with greater distribution ranges that extend from warm-temperate to tropical regions, which could be related to their longer evolutionary history; whereas the later appeared ER-type species have smaller distribution ranges as they are absent in subtropical climates. The thermal distribution centers based on mean and median annual temperatures were consistent with each other (Table 3 & S4), which further supports the tropical and warm temperate speciation centers. Combined with previous phylogenetic results, we conclude that the ER fruit type has evolved independently multiple times across various lineages in both tropical and subtropical-warm temperate regions.

As we found ER-type *Lithocarpus* species to be polyphyletic, environment could be a plausible driver for the fruit type evolution and differentiation in the genus. Evolution of fruits mirrors the co-evolutionary history between fruits and animals. During seed predation and dispersal, fruits associated with insects represent antagonism, whereas those associated with vertebrates represent mutualism [28]. Pre-dispersal seed predation by insects causes seed death or damage, which negatively impacts seed dispersal, germination, and successful seedling establishment [28, 31, 32]. On the other hand, vertebrates such as rodents are effective seed dispersers as their forgetfulness could reduce seed consumption and enhance seed germination as well as seedling establishment [5, 17, 20, 33]. The various fruit characteristics, i.e., morphology, seed chemistry, and physical defense, act as an entity interacting with their predators and dispersers, which shapes the co-evolution between fruits and animals [7–12]. The predation selection hypothesis proposes that the morphological and mechanical trade-off between the two fruit types could be results under different predation pressure: smaller-sized AC fruits attract a wider range of dispersers, but their weaker physical defense could cause higher pre-dispersal predation, whereas ER fruits with thicker husk could inhibit insect infestation more effectively, but their larger fruit could only be dispersed by larger-body sized vertebrates [26]. The mechanical trade-off between AC and ER fruit types that we identified partially supports the predation selection hypothesis, but the variation in predation and dispersal between the two fruit types is still the missing link. The common insect herbivores of both fruit types of *Lithocarpus* are weevils, gall wasps, bark beetles, and crane flies [16, 17], but whether there is a difference in pre-dispersal predation between the two fruit types is yet to be examined. In terms of the seed dispersers, while rats and squirrels are known to disperse AC fruits [18–20], the species that disperse ER fruits are currently not well documented. Thus, to further test the predation selection hypothesis, a detailed study of the insect and vertebrate predation and seed dispersal of AC and ER fruits is crucial in clarifying the predation selection hypothesis in the future.

## Conclusion

Overall, our study provides important information for understanding the fruit morphometric and fruit type evolution of stone oaks. We examined the morphological and mechanical trade-off between the two fruit types: the larger seeds of ER fruits are mainly enclosed by receptacle tissue representing stronger physical defense, whereas the smaller seeds of AC fruits are enclosed by thin pericarp representing lower physical protection. The mechanical trade-off between the two fruit types serves as evidence for the predation selection hypothesis. The phylogenetic analysis supports the hypothesis that ER is the derived fruit type from AC-like ancestors independently across all clades. Instead of directional selection of ER species [24], we propose a divergent selection theory for two fruit types: the seed size and mechanical defense of AC fruits were reduced, whereas those of ER fruits increased and required more morphological modifications in receptacle through evolutionary time. This result signifies the important role of the receptacle in the fruit type evolution. Lastly, we found that the phylogenetic radiation of *Lithocarpus* involved two geographical speciation centers: tropical and subtropical-warm temperate regions. As ER fruits are results of convergent evolution, we propose to examine whether there are different types of predation pressure acting as drivers for the evolution of the two fruit types across climatic regions in the future.

## Methods

### Fruit morphometrics data collection

#### Sampling design and fruit image preparation

In total, we examined the morphometrics of 2,613 mature fruits of 168 species, including 98 species and 595 fruit samples which were applied in the previous fruit morphometric study [26]. The 168 species represent about half of the recorded *Lithocarpus* species and encompass a wide range of morphological variation and geographic distribution (Table 1). The majority of the fruit specimens were collections from six herbaria: the National Herbarium Netherlands, the Harvard University Herbaria, the Herbarium of Kunming Institute of Botany of the Chinese Academy of Sciences, Smithsonian National Museum of Natural History, US National Herbarium, the VNM Herbarium, and the Herbarium of Kyushu University. Additional fruit specimens came from our field collections from southern China between 2015 and 2020. We confirmed the nomenclatures and corrected synonyms based on the International Plant Names Index (2019, http://ipni.org).

Fruit dissection followed the protocol of our previous morphometric study [26], and the images of fruit longitudinal section were captured with a Canon SLR camera (EOS M3, Tokyo, Japan). The fruit image standardization and processing was performed in Adobe Photoshop CS 5.1 [26].

#### Fruit morphometric dimensions’ estimation

Assuming each fruit to be a perfect rotating body, we employed the Pappus-Guldinus Theorem to reconstruct 3D fruit shapes from the 2D fruit images [17, 26] and to obtain six fruit dimensions S_s_, S_r_, S_p_, V_s_, V_r_ and V_p_, i.e., the coverage (S) and the volume (V) of seed space (s), receptacle (r), and pericarp (p) respectively. To estimate these six fruit dimensions, 12 parameters of each fruit were collected using Image J 1.51 h [34]: the internal curve length of pericarp (L_p_), internal curve length of receptacle (L_r_), curve length of seed space (L_s_), and the distances from longitudinal axis to the centroid of L_p_, L_r_ and L_s_, namely r_p_, r_r_, and r_s_, respectively (Fig. 1b & e); the section area of pericarp (A_p_), receptacle (A_r_), and seed space (A_s_), the distances from the longitudinal axis to the centroid of A_p_, A_r_, and A_s_, namely R_p_, R_r_ and R_s_, respectively (Fig. 1c & f). The surface area of the seed space (S_s_), the coverage by pericarp (S_p_), the coverage by receptacle (S_r_), and volume of pericarp (V_p_), receptacle (V_r_) and seed space (V_s_) were determined by the Pappus-Guldinus Theorem [26]. The fruit husk volume was defined as V_h_ = V_p_ + V_r_.

#### Fruit type identification and fruit morphometric analysis

All 168 species were classified into 138 AC- and 30 ER-type species based on the species average seed surface coverage by pericarp and receptacle (Table 1) [27]. The marked difference between pericarp and receptacle coverage (Table 1) of 23 species with a single fruit sample indicated a clear fruit type distinction.

The morphometric analysis was performed in R 4.1.0 [35]. We hypothesized a morphometric trade-off and mechanical distinction between AC- and ER-type species: smaller-seeded AC species were mainly enclosed by pericarp which represents lower mechanical defense, whereas larger-seeded ER species were mainly enclosed by receptacle representing better mechanical defense. To test this hypothesis, we first applied two linear mixed models (package lme 4 [36]) with Gaussian-distributed errors to examine the variation in mechanical defense (fruit husk volume divided by seed surface area) and seed volume between the two fruit types by employing all 168 species. Model one set fruit type as the fixed factor, species as random factors; model two only included species as the random factors. We selected the best model by comparing two models by Akaike information criterion (AIC). Then, we employed the standard major axis (MA) regression with package smatr 3.4.3 [37] to compare the seed enclosure by pericarp and receptacle within each fruit type and analyze intra- and inter-specific allometry across selected pairs of dimensions after logarithmic transformation. Interspecific variation was examined on 168 species represented by 2,613 fruit samples. Intraspecific variation was examined on 61 species with eight or more fruit samples each, including 11 ER-type and 50 AC-type species with a total of 2,254 fruit samples.

### Phylogenetic and combined morphometric analysis

Yang et al. [30] used cpDNA (*atp*B-*rbc*L) and nrITS of 64 species to reconstruct the phylogenetic relationship of *Lithocarpus*. Here, we retrieved one nuclear (nrITS) and five chloroplast gene fragments (*atp*B-*rbc*L, *mat*K, *rbc*L, *trn*L-*trn*F, *psb*A- *trn*H) of 72 out of the 168 study species from NCBI Genbank (https://www.ncbi.nlm.nih.gov). Sequences were aligned by MEGA-X v.10.2.2 [38] and manually edited using SequenceMatrix [39]. Individual gene alignments were concatenated into different data sets to reconstruct nrITS, cpDNA, and nrITS + cpDNA phylogenies, respectively (Table S1 & S2). We used *Chrysolepis chrysophylla* as an outgroup to root the tree.

Partitioned Bayesian and Maximum Likelihood (ML) analyses were then conducted on the three concatenated data sets. Bayesian inference was performed in MrBayes v.3.2.7 [40]. According to the Akaike Information Criterion (AIC) values obtained using ModelFinder [41], the best-fit models of nucleotide substitution for the *atp*B-*rbc*L, *psb*A-*trn*H, *mat*K, *rbc*L, *trn*L-*trn*F and nrITS datasets were determined to be TIM3 + I, TIM3 + I, TVM, GTR, TPM1uf and GTR + G, respectively. Two independent runs with one cold and three incrementally heated Monte Carlo Markov chains (MCMCs) were run for 50,000,000 generations, with trees sampled every 500th generation. Model parameters were unlinked across partitions. We discarded the first 2,500 trees out of the 10,001 trees as burn-ins and used the remaining trees to build a 50% majority rule consensus tree. Maximum likelihood analyses were performed using raxmlGUI 2.0 [42]. A separate General Time Reversible + Gamma model (GTR + G) of nucleotide substitution was specified for each data partition, and 500 independent searches were conducted. Support values for nodes in the phylogenetic tree were estimated across 1,000 pseudoreplicates using the GTRGAMMA model and mapped thereafter onto the best-scoring tree from the 500 independent searches. Finally, FigTree v1.4.4 (http://tree.bio.ed.ac.uk/software/figtree) was applied to visualize the phylogenetic trees.

Based on the phylogeny reconstructed from the concatenated data sets, we ran RASP v.4 [43, 44] and Mesquite v.3.61[45] to infer the ancestral states and evolution of the fruit type and the six fruit morphometric dimensions (S_p_, S_r_, V_P_, V_R_, V_H_ and V_S,_ after log-transformation), respectively. The phylogenetic signals of the fruit types and six fruit morphometric measures were also estimated in RASP v.4. For comparative purpose, these analyses were also conducted based on the phylogenetic tree of 64 *Lithocarpus* species that was reconstructed by Yang et al. [30] using cpDNA and nrITS data.

### Thermal distribution analysis

The morphometrics of both fruit types was found positively related to the mean annual temperature of 91 Chinese *Lithocarpus* species in our previous study [27]. To identify the thermal distribution differences among lineages with AC- and ER-type species, we retrieved the present-day distribution ranges and location description of 72 species from the Plants of the World Online website (https://powo.science.kew.org/), then applied Google Earth (https://www.google.com/earth/) to obtain the geographic coordinates for each locality. To better match with the land size of Southeast Asian countries, the location resolution in China was set to provincial level. As the morphometrics of both AC and ER species were positively related to mean annual temperature according to our previous study [27], we applied Raster v3.4.10 [46] to obtain mean annual temperature (MAT; in degree Celsius) from WorldClim 2.0 (https://www.worldclim.org/). The median annual temperature among distribution locations were calculated for each species, and the thermal distribution was categorized as follows: (i) MAT > 22 °C, tropical; (ii) MAT in between 19 °C and 22 °C, subtropical; (iii) MAT in between 11 °C and 19 °C, warm temperate to correspond to the warmth-index based definition of our target regions [47].

## Supplementary Information

**Additional file 1: Figure S1.** Fruit morphologies of the two exceptional species with negative allometric slopes. (a)-(c), ER-type species, *L. javensis*. (d)–(f), AC-type species, *L. ferrugineus*. Pericarp and receptacle tissues were depicted by solid red lines and dashed green lines on the left side of the longitudinal section respectively.

**Additional file 2: Figure S2.** The ML phylogenetic trees. (a) The ML phylogenetic tree based on cpDNA of 58 species. (b) The ML phylogenetic tree based on nrITS of 66 species.

**Additional file 3: Figure S3.** The Bayesian phylogenetic trees are based on cpDNA + nrITS of 72 species.

**Additional file 4: Figure S4.** Matching the fruit type to the phylogenetic tree proposed by Yang et. al (2018). Based on the cpDNA + nrITS phylogenetic tree (Fig. 2a) by Yang et al, AC and ER type species were represented by red circles and blue triangles after the species name respectively. The species with unidentified fruit type was not labelled.

**Additional file 5: Figure S5.** Ancestral state reconstruction of pericarp and receptacle volume of 72 *Lithocarpus* species. Estimated ancestral morphometric values are coded by colored branches as explained in the legend within the figure. The species names of AC and ER-type species are colored by black and green respectively.

**Additional file 6: Figure S6.** The four species exhibiting AC-ER intermediate fruit morphology. (a) *L. pachylepis*. (b) *L. lampadarius*. (c) *L. revolutus*. (d) *L. pulcher*) all represent a similar fruit morphology with unreduced or thickened pericarp (red solid line) and extended and thickened receptacle (green dashed line).

**Additional file 7: Table S1.** The genes fragments and accession number of 72 *Lithocarpus* species and *Chrysolepis chrysophylla* applied in the phylogenetic study.

**Additional file 8: Table S2.** The comparison of species included in our phylogenetic study and study by Yang et al. (2018).

**Additional file 9: Table S3.** The six fruit morphometrics estimated by Pappus-Guldinus Theorem.

**Additional file 10: Table S4.** The distribution of 72 species from the plants of the world online (https://powo.science.kew.org/).

**Additional file 11: Table S5.** The deposition numbers of the dissected fruit samples from the herbarium specimens.

## Data Availability

The fruit morphometric dataset supporting the conclusions of this article is included within the article (Table 1). Sequencing data are deposited in Sequence Read Archive of NCBI (https://www.ncbi.nlm.nih.gov), the accession numbers are listed in Table S1.

## References

[1] Vander Wall SB, Beck SB (2012). A comparison of frugivory and scatter-hoarding seed-dispersal syndromes on JSTOR. Bot Rev.

[2] Forget PM, Hammond DS, Milleron T, Thomas R. Seasonality of fruiting and food hoarding by rodents in neotropical forests: consequences for seed dispersal and seedling recruitment. Seed dispersal frugivory Ecol Evol Conserv Third Int Symp Frugivores Seed Dispersal, São Pedro, Brazil, 6-11 August 2000. 2002;241–56.

[3] Foster SA (1986). On the adaptive value of large seeds for tropical moist forest trees: a review and synthesis. Bot Rev.

[4] Morley RJ (2001). Why are there so many primitive Angiosperms in the rain forests of Asia-Australasia?. Faunal and Floral Migrations and Evolution in Se Asia-Australasia.

[5] Vander Wall SB (2001). The evolutionary ecology of nut dispersal. Bot Rev.

[6] Eriksson O (2016). Evolution of angiosperm seed disperser mutualisms: the timing of origins and their consequences for coevolutionary interactions between angiosperms and frugivores. Biol Rev.

[7] Janzen DH (1971). Seed predation by animals. Annu Rev Ecol Syst.

[8] Xiao Z, Harris MK, Zhang Z (2007). Acorn defenses to herbivory from insects: implications for the joint evolution of resistance, tolerance and escape. For Ecol Manage.

[9] Norconk MA, Grafton BW, Conklin-Brittain NL (1998). Seed dispersal by neotropical seed predators. Am J Primatol.

[10] Wang B, Chen J (2009). Seed size, more than nutrient or tannin content, affects seed caching behavior of a common genus of Old World rodents. Ecology.

[11] Wang B, Yang X (2014). Teasing apart the effects of seed size and energy content on rodent scatter-hoarding behavior. PLoS One.

[12] Wang B, Yang X (2015). Seed removal by scatter-hoarding rodents: the effects of tannin and nutrient concentration. Behav Processes.

[13] Camus A (1952). Les chênes: monographie du genre *Quercus*.

[14] van Soepadmo E. SC (1972). Fagaceae. Flora Malesiana-Series 1, Spermatophyta.

[15] Huang C j, Zhang Y t, Bartholomew B (2000). Fagaceae. Flora of China: Cycadaceae through Fagaceae.

[16] Teruya K, Shinzato T, Sasaki T, Nakao T (2010). Annual fluctuation and seasonal falling pattern of mature acorns of the beech family and a survey of their acorn-infesting insect fauna on subtropical Okinawa Island. Tropics.

[17] Chen X, Kohyama TS, Cannon CH (2020). Fruit development of *Lithocarpus* (Fagaceae) and the role of heterochrony in their evolution. J Plant Res.

[18] Xiao Z, Zhang Z, Wang Y (2003). Observations on tree seed selection and caching by Edward’s long-tailed rat (*Leopoldamys edwardsi*) (in Chinese). Acta Theriol Sin.

[19] Zhang T, Li K, Cai Y, Yang K, Hu Xi, Peng S (2006). Predation and dispersal of *Lithocarpus glaber* seeds by rodents in Tiantong National Forest Park, Zhejiang province (in Chinese). Chinese J Appl Ecol.

[20] Xiao Z, Zhang Z (2006). Nut predation and dispersal of harland tanoak *Lithocarpus harlandii* by scatter-hoarding rodents. Acta Oecologica.

[21] Brett DW (1964). The inflorescence of *Fagus* and *Castanea*, and the evolution of the cupules of the Fagaceae. New Phytol.

[22] Fey BS, Endress PK (1983). Development and morphological interpretation of the cupule in Fagaceae. Flora.

[23] Kaul RB (1987). Reproductive structure of *Lithocarpus* sensu lato (Fagaceae): cymules and fruits. J Arnold Arbor.

[24] Cannon CH, Manos PS (2001). Combining and comparing morphometric shape descriptors with a molecular phylogeny: the case of fruit type evolution in Bornean *Lithocarpus* (Fagaceae). Syst Biol.

[25] Chen X, Cannon CH, Conklin-Brittan N Lou (2012). Evidence for a trade-off strategy in stone oak (*Lithocarpus*) seeds between physical and chemical defense highlights fiber as an important antifeedant. PLoS One..

[26] Chen X, Kohyama TS, Cannon CH (2018). Associated morphometric and geospatial differentiation among 98 species of stone oaks (*Lithocarpus*). PLoS One.

[27] Chen X, Kohyama TS (2022). Variation among 91 stone oak species (Fagaceae, Lithocarpus) in fruit and vegetative morphology in relation to climatic factors. Flora.

[28] Crawley MJ (2000). Seed predators and plant population dynamics. Seeds: the ecology of regeneration in plant communities.

[29] Zhou BF, Yuan S, Crowl AA, Liang YY, Shi Y, Chen XY (2022). Phylogenomic analyses highlight innovation and introgression in the continental radiations of Fagaceae across the Northern Hemisphere. Nat Commun.

[30] Yang C-K, Chiang Y-C, Huang B-H, Ju L-P, Liao P-C (2018). Nuclear and chloroplast DNA phylogeography suggests an Early Miocene southward expansion of *Lithocarpus* (Fagaceae) on the Asian continent and islands. Bot Stud.

[31] Crawley MJ, Long CR (1995). Alternate bearing, predator satiation and seedling recruitment in *Quercus Robur* L. J Ecol.

[32] Xiao Z, Zhang Z, Wang Y (2003). Rodent’s ability to discriminate weevil-infested acorns: potential effects on regeneration of nut-bearing plants. Acta Theriol Sin.

[33] Iseki N, Sasaki A, Toju H (2011). Arms race between weevil rostrum length and camellia pericarp thickness: geographical cline and theory. J Theor Biol.

[34] Schneider CA, Rasband WS, Eliceiri KW (2012). NIH Image to ImageJ: 25 years of image analysis. Nat Methods.

[35] R Core Team. 2019.

[36] Douglas A , Walker S, Singmann H, Dai B, Ben M (2017). Ben M. Package ‘lme4'.

[37] Warton DI, Duursma RA, Falster DS, Taskinen S (2012). Smatr 3- an R package for estimation and inference about allometric lines. Methods Ecol Evol.

[38] Sudhir K, Glen S, Michael L, Christina K, Koichiro T (2018). MEGA X: molecular evolutionary genetics analysis across computing platforms. Mol Biol Evol.

[39] Vaidya G, Lohman DJ, Meier R (2011). SequenceMatrix: concatenation software for the fast assembly of multi-gene datasets with character set and codon information. Cladistics.

[40] Ronquist F, Teslenko M, van der Mark P, Ayres DL, Darling A, Höhna S (2012). MrBayes 3.2: efficient Bayesian phylogenetic inference and model choice across a large model space. Syst Biol..

[41] Kalyaanamoorthy S, Minh BQ, Wong TKF, von Haeseler A, Jermiin LS (2017). ModelFinder: fast model selection for accurate phylogenetic estimates. Nat Methods.

[42] Edler D, Klein J, Antonelli A, Silvestro D (2021). raxmlGUI .2.0: a graphical interface and toolkit for phylogenetic analyses using RAxML. Methods Ecol Evol.

[43] Yu Y, Harris AJ, Blair C, He X (2015). RASP (Reconstruct Ancestral State in Phylogenies): a tool for historical biogeography. Mol Phylogenet Evol.

[44] Yu Y, Blair C, He X (2020). RASP 4: ancestral state reconstruction tool for multiple genes and characters. Mol Biol Evol.

[45] Maddison WP, Maddison DR (2019). Mesquite: a modular system for evolutionary analysis.

[46] Hijmans RJ, van Etten J (2014). raster: Geographic data analysis and modeling (R package).

[47] Kira T (1991). Forest ecosystems of east and southeast Asia in a global perspective. Ecol Res.

