# Supplementary material for: The fruit morphometric variation and fruit type evolution of the stone oaks (Fagaceae, Lithocarpus)
Source: BMC Plant Biol. 2023 Apr 29;23:229. doi: 10.1186/s12870-023-04237-4 (PMC10148511; doi:10.1186/s12870-023-04237-4)
Supplement: Supplementary file 1 — Additional file 1: Figure S1. Fruit morphologies of the two exceptional species with negative allometric slopes. (a)-(c), ER-type species, L. javensis. (d)–(f), AC-type species, L. ferrugineus. Pericarp and receptacle tissues were depicted by solid red lines and dashed green lines on the left side of the longitudinal section respectively. [file 12870_2023_4237_MOESM1_ESM.docx]

Table S4 The distribution of 72 species from the plants of the world online (<https://powo.science.kew.org/>)

| Fruit type | species | Distribution |  |  |  |  |  | |  |  |  |  |  |  |  |  |  |  |  |  |
| --- | --- | --- | --- | --- | --- | --- | --- | --- | --- | --- | --- | --- | --- | --- | --- | --- | --- | --- | --- | --- |
| AC | *L. bancanus* | Borneo | Malaya | Sumatera | Thailand |  |  | |  |  |  |  |  |  |  |  |  |  |  |  |
| AC | *L. bennettii* | Borneo | Malaya | Sumatera | Thailand |  |  |  | |  |  |  |  |  |  |  |  |  |  |  |
| AC | *L. blumeanus* | Borneo | Myanmar | Thailand |  |  |  |  | |  |  |  |  |  |  |  |  |  |  |  |
| AC | *L. brevicaudatus* | Hainan | Taiwan | Yunnan | Sichuan | Guizhou | Chongqing | Guangxi | | Guangdong | Fujian | Jiangxi | Hunan | Zhejiang |  |  |  |  |  |  |
| AC | *L. calophyllus*** | Fujian | Guangdong | Guangxi | Guizhou | Hunan | Jiangxi |  | |  |  |  |  |  |  |  |  |  |  |  |
| AC | *L. cantleyanus* | Borneo | Malaya | Myanmar | Thailand |  |  |  | |  |  |  |  |  |  |  |  |  |  |  |
| AC | *L. chrysocomus** | Guangdong | Guangxi | Hunan |  |  |  |  | |  |  |  |  |  |  |  |  |  |  |  |
| AC | *L. clementianus* | Borneo | Malaya | Thailand |  |  |  |  | |  |  |  |  |  |  |  |  |  |  |  |
| AC | *L. conocarpus* | Borneo | Jawa | Malaya | Sumatera |  |  |  | |  |  |  |  |  |  |  |  |  |  |  |
| AC | *L. cooperatus* | Borneo | Malaya | Philippines |  |  |  |  | |  |  |  |  |  |  |  |  |  |  |  |
| AC | *L. dasystachyus* | Borneo |  |  |  |  |  |  | |  |  |  |  |  |  |  |  |  |  |  |
| AC | *L. dealbatus** | Assam | Bangladesh | Yunnan | East Himalaya | Laos | Myanmar | Nepal | | Thailand | Vietnam |  |  |  |  |  |  |  |  |  |
| AC | *L. echinophorus** | Yunnan | Thailand | Vietnam |  |  |  |  | |  |  |  |  |  |  |  |  |  |  |  |
| AC | *L. echinotholus* | Vietnam | Yunnan |  |  |  |  |  | |  |  |  |  |  |  |  |  |  |  |  |
| AC | *L. edulis* | Japan | Nansei-shoto |  |  |  |  |  | |  |  |  |  |  |  |  |  |  |  |  |
| AC | *L. elegans** | Assam | Bangladesh | Borneo | Cambodia | Yunnan | Sichuan | Guizhou | | East Himalaya | Jawa | Laos | Malaya | Myanmar | Nepal | Sulawesi | Sumatera | Thailand | Tibet | Vietnam |
| AC | *L. encleisocarpus* | Siam | Sumatera | Malaya | Borneo |  |  |  | |  |  |  |  |  |  |  |  |  |  |  |
| AC | *L. ewyckii* | Borneo | Malaya | Sumatera |  |  |  |  | |  |  |  |  |  |  |  |  |  |  |  |
| AC | *L. fenestratus* | Assam | Bangladesh | Yunnan | Sichuan | Guizhou | Chongqing | Guangxi | | Guangdong | Fujian | Jiangxi | Hunan | Hainan | Laos | Myanmar | Nepal | Thailand | Tibet | Vietnam |
| AC | *L. ferrugineus* | Borneo |  |  |  |  |  |  | |  |  |  |  |  |  |  |  |  |  |  |
| AC | *L. formosanus* | Taiwan |  |  |  |  |  |  | |  |  |  |  |  |  |  |  |  |  |  |
| AC | *L. gigantophyllus* | Cambodia | Vietnam |  |  |  |  |  | |  |  |  |  |  |  |  |  |  |  |  |
| AC | *L. glaber* | Japan | Nansei-shoto | Yunnan | Sichuan | Guizhou | Chongqing | Guangxi | | Guangdong | Fujian | Jiangxi | Hunan | Taiwan |  |  |  |  |  |  |
| AC | *L. gracilis* | Borneo | Malaya | Sumatera |  |  |  |  | |  |  |  |  |  |  |  |  |  |  |  |
| AC | *L. grandifolius** | Assam | Bangladesh | Borneo | Cambodia | Yunnan | Sichuan | Guizhou | | East Himalaya | Jawa | Laos | Malaya | Myanmar | Nepal | Sulawesi | Sumatera | Thailand | Tibet | Vietnam |
| AC | *L. hancei* | Yunnan | Sichuan | Guizhou | Chongqing | Guangxi | Guangdong | Fujian | | Jiangxi | Hunan | Taiwan |  |  |  |  |  |  |  |  |
| AC | *L. handelianus* | Hainan |  |  |  |  |  |  | |  |  |  |  |  |  |  |  |  |  |  |
| AC | *L. harlandii* | Yunnan | Sichuan | Guizhou | Chongqing | Guangxi | Guangdong | Fujian | | Jiangxi | Hunan | Taiwan |  |  |  |  |  |  |  |  |
| AC | *L. henryi* | Hubei | Sichuan | Guizhou | Hunan | Jiangsu | Shanxi |  | |  |  |  |  |  |  |  |  |  |  |  |
| AC | *L. jacobsii* | Borneo |  |  |  |  |  |  | |  |  |  |  |  |  |  |  |  |  |  |
| AC | *L. kawakamii* | Taiwan |  |  |  |  |  |  | |  |  |  |  |  |  |  |  |  |  |  |
| AC | *L. konishii* | Hainan | Taiwan |  |  |  |  |  | |  |  |  |  |  |  |  |  |  |  |  |
| AC | *L. leptogyne* | Borneo | Malaya | Sumatera |  |  |  |  | |  |  |  |  |  |  |  |  |  |  |  |
| AC | *L. licentii* | Vietnam |  |  |  |  |  |  | |  |  |  |  |  |  |  |  |  |  |  |
| AC | *L. lindleyanus* | Bangladesh | Cambodia | Myanmar | Thailand | Vietnam |  |  | |  |  |  |  |  |  |  |  |  |  |  |
| AC | *L. litseifolius* | Assam | Yunnan | Sichuan | Guizhou | Chongqing | Guangxi | Guangdong | | Fujian | Jiangxi | Hunan | Hainan | Laos | Myanmar | Vietnam |  |  |  |  |
| AC | *L. longipedicellatus** | Yunnan | Guangxi | Hainan | Vietnam |  |  |  | |  |  |  |  |  |  |  |  |  |  |  |
| AC | *L. lucidus* | Borneo | Malaya | Sumatera | Thailand |  |  |  | |  |  |  |  |  |  |  |  |  |  |  |
| AC | *L. luteus* | Borneo |  |  |  |  |  |  | |  |  |  |  |  |  |  |  |  |  |  |
| AC | *L. mairei* | Yunnan |  |  |  |  |  |  | |  |  |  |  |  |  |  |  |  |  |  |
| AC | *L. meijeri* | Borneo |  |  |  |  |  |  | |  |  |  |  |  |  |  |  |  |  |  |
| AC | *L. naiadarum* | Hainan |  |  |  |  |  |  | |  |  |  |  |  |  |  |  |  |  |  |
| AC | *L. nieuwenhuisii* | Borneo | Philippines |  |  |  |  |  | |  |  |  |  |  |  |  |  |  |  |  |
| AC | *L. pachyphyllus* | Assam | Bangladesh | Yunnan | Sichuan | Guizhou | Chongqing | Guangxi | | Guangdong | Fujian | Jiangxi | Hunan | Myanmar | Nepal | Tibet |  |  |  |  |
| AC | *L. rosthornii* | Sichuan | Guizhou | Guangdong | Guangxi |  |  |  | |  |  |  |  |  |  |  |  |  |  |  |
| AC | *L. rufovillosus* | New Guinea |  |  |  |  |  |  | |  |  |  |  |  |  |  |  |  |  |  |
| AC | *L. sericobalanos* | Borneo |  |  |  |  |  |  | |  |  |  |  |  |  |  |  |  |  |  |
| AC | *L. shinsuiensis* | Taiwan |  |  |  |  |  |  | |  |  |  |  |  |  |  |  |  |  |  |
| AC | *L. silvicolarum* | Assam | Yunnan | Sichuan | Guizhou | Chongqing | Guangxi | Guangdong | | Fujian | Jiangxi | Hunan | Hainan | Laos | Myanmar | Vietnam |  |  |  |  |
| AC | *L. skanianus* | Yunnan | Fujian | Guangdong | Guangxi | Hainan | Hunan | Jiangxi | |  |  |  |  |  |  |  |  |  |  |  |
| AC | *L. stenopus* | Vietnam |  |  |  |  |  |  | |  |  |  |  |  |  |  |  |  |  |  |
| AC | *L. taitoensis* | Yunnan | Sichuan | Guizhou | Chongqing | Guangxi | Guangdong | Fujian | | Jiangxi | Hunan | Taiwan |  |  |  |  |  |  |  |  |
| ER | *L. amygdalifolius** | Guangdong | Fujian | Hainan | Taiwan | Vietnam |  |  | |  |  |  |  |  |  |  |  |  |  |  |
| ER | *L. balansae** | Yunnan | Laos | Myanmar | Vietnam |  |  |  | |  |  |  |  |  |  |  |  |  |  |  |
| ER | *L. beccarianus* | Borneo |  |  |  |  |  |  | |  |  |  |  |  |  |  |  |  |  |  |
| ER | *L. cleistocarpus* | Yunnan | Sichuan | Guizhou | Chongqing | Guangxi | Guangdong | Fujian | | Jiangxi | Hunan | Zhejiang |  |  |  |  |  |  |  |  |
| ER | *L. corneus*** | Yunnan | Sichuan | Guizhou | Chongqing | Guangxi | Guangdong | Fujian | | Jiangxi | Hunan | Zhejiang | Hainan | Laos | Taiwan | Thailand | Vietnam |  |  |  |
| ER | *L. echinifer* | Borneo |  |  |  |  |  |  | |  |  |  |  |  |  |  |  |  |  |  |
| ER | *L. fenzelianus* | Hainan |  |  |  |  |  |  | |  |  |  |  |  |  |  |  |  |  |  |
| ER | *L. kalkmanii* | Borneo |  |  |  |  |  |  | |  |  |  |  |  |  |  |  |  |  |  |
| ER | *L. lampadarius* | Borneo | Malaya |  |  |  |  |  | |  |  |  |  |  |  |  |  |  |  |  |
| ER | *L. laoticus* | Yunnan | Sichuan | Chongqing | Hubei | Laos | Tibet | Vietnam | |  |  |  |  |  |  |  |  |  |  |  |
| ER | *L. lepidocarpus* | Taiwan |  |  |  |  |  |  | |  |  |  |  |  |  |  |  |  |  |  |
| ER | *L. pachylepis** | Yunnan | Guangxi | Vietnam |  |  |  |  | |  |  |  |  |  |  |  |  |  |  |  |
| ER | *L. pulcher* | Borneo |  |  |  |  |  |  | |  |  |  |  |  |  |  |  |  |  |  |
| ER | *L. revolutus* | Borneo | Thailand |  |  |  |  |  | |  |  |  |  |  |  |  |  |  |  |  |
| ER | *L. rumitus* | Borneo |  |  |  |  |  |  | |  |  |  |  |  |  |  |  |  |  |  |
| ER | *L. truncatus** | Assam | Bangladesh | Yunnan | Sichuan | Chongqing | Laos | Myanmar | | Thailand | Tibet | Vietnam |  |  |  |  |  |  |  |  |
| ER | *L. turbitus* | Borneo |  |  |  |  |  |  | |  |  |  |  |  |  |  |  |  |  |  |
| ER | *L. uvariifolius* | Guangxi | Guangdong | Jiangxi | Fujian | Zhejiang | Anhui | Hubei | | Shanghai |  |  |  |  |  |  |  |  |  |  |
| ER | *L. variolosus* | Sichuan | Yunnan |  |  |  |  |  | |  |  |  |  |  |  |  |  |  |  |  |
| ER | *L. xylocarpus** | Assam | Bangladesh | Yunnan | Sichuan | Chongqing | Laos | Myanmar | | Thailand | Tibet | Vietnam |  |  |  |  |  |  |  |  |

One aster mark (*) indicated that thermal region identified based on median MAT is hotter than that of mean MAT; two aster mark (**) indicated that thermal region identified based on mean MAT is hotter than that of median MAT.
