# Supplementary material for: The fruit morphometric variation and fruit type evolution of the stone oaks (Fagaceae, Lithocarpus)
Source: BMC Plant Biol. 2023 Apr 29;23:229. doi: 10.1186/s12870-023-04237-4 (PMC10148511; doi:10.1186/s12870-023-04237-4)
Supplement: Supplementary file 2 — Additional file 2: Figure S2. The ML phylogenetic trees. (a) The ML phylogenetic tree based on cpDNA of 58 species. (b) The ML phylogenetic tree based on nrITS of 66 species. [file 12870_2023_4237_MOESM2_ESM.docx]

Table S5 The deposition numbers of the dissected fruit samples from the herbarium specimens

| Species | Herbaria | Deposition number |
| --- | --- | --- |
| *L. amygdalifolius* | The Harvard University Herbaria | 10891 |
| *L. bacgiangensis* | The Harvard University Herbaria | 11596 |
| *L. bacgiangensis* | The Harvard University Herbaria | 78444 |
| *L. bacgiangensis* | The Herbarium of Kunming Institute of Botany of the Chinese Academy of Sciences | 500884 |
| *L. balansae* | The Herbarium of Kunming Institute of Botany of the Chinese Academy of Sciences | 500911 |
| *L. bancanus* | The National Herbarium Netherlands | B-102 |
| *L. beccarianus* | The National Herbarium Netherlands | 99207 |
| *L. bennettii* | The National Herbarium Netherlands | Ashton5236 |
| *L. bennettii* | The National Herbarium Netherlands | IliasPaieS.15878 |
| *L. bennettii* | The National Herbarium Netherlands | JugahS.23688 |
| *L. bennettii* | The National Herbarium Netherlands | Wiriandinata3529 |
| *L. bennettii* | The National Herbarium Netherlands | Wood&WyattSmithA.4569 |
| *L. blumeanus* | The National Herbarium Netherlands | Arsat1150 |
| *L. blumeanus* | The National Herbarium Netherlands | Koorders33442 |
| *L. blumeanus* | The National Herbarium Netherlands | 34/1-35 |
| *L. blumeanus* | The National Herbarium Netherlands | Wood&Forest16199 |
| *L. brachystachyus* | The Herbarium of Kunming Institute of Botany of the Chinese Academy of Sciences | 500926 |
| *L. brevicaudatus* | The Harvard University Herbaria | 3637 |
| *L. calophyllus* | The Herbarium of Kunming Institute of Botany of the Chinese Academy of Sciences | 804301 |
| *L. carolinae* | The Herbarium of Kunming Institute of Botany of the Chinese Academy of Sciences | 500952 |
| *L. caudatilimbus* | The Herbarium of Kunming Institute of Botany of the Chinese Academy of Sciences | 500951 |
| *L. celebicus* | The National Herbarium Netherlands | A.G.Waturaudaug25.1934 |
| *L. celebicus* | The National Herbarium Netherlands | H.N.Reppie159.1934 |
| *L. celebicus* | The National Herbarium Netherlands | Koorders16622 |
| *L. chrysocomus* | The Herbarium of Kunming Institute of Botany of the Chinese Academy of Sciences | 500958 |
| *L. cleistocarpus* | The Herbarium of Kunming Institute of Botany of the Chinese Academy of Sciences | 500987 |
| *L. cleistocarpus* | The Herbarium of Kunming Institute of Botany of the Chinese Academy of Sciences | 500971 |
| *L. cleistocarpus* | The Herbarium of Kunming Institute of Botany of the Chinese Academy of Sciences | 501036 |
| *L. cleistocarpus* | The Harvard University Herbaria | 728 |
| *L. cleistocarpus* | The Harvard University Herbaria | 4009 |
| *L. confertus* | The National Herbarium Netherlands | J.&M.S.Clemens29265 |
| *L. confinis* | The Herbarium of Kunming Institute of Botany of the Chinese Academy of Sciences | 501068 |
| *L. confinis* | The Herbarium of Kunming Institute of Botany of the Chinese Academy of Sciences | 501059 |
| *L. confinis* | The Harvard University Herbaria | 34945 |
| *L. conocarpus* | The National Herbarium Netherlands | H.Hallier2518 |
| *L. corneus* | The Herbarium of Kunming Institute of Botany of the Chinese Academy of Sciences | 501163 |
| *L. corneus* | Smithsonian National Museum of Natural History | 337 |
| *L. corneus* | The Harvard University Herbaria | 70116 |
| *L. corneus* | The Harvard University Herbaria | 18054 |
| *L. corneus* | The Harvard University Herbaria | 18.271 |
| *L. corneus* | The Harvard University Herbaria | 24159 |
| *L. corneus* | The Harvard University Herbaria | 8523 |
| *L. corneus* | The Harvard University Herbaria | 24803 |
| *L. corneus* | The Harvard University Herbaria | 24159 |
| *L. corneus* | The Harvard University Herbaria | 8523 |
| *L. corneus* | The Herbarium of Kunming Institute of Botany of the Chinese Academy of Sciences | 501163 |
| *L. craibianus* | The Herbarium of Kunming Institute of Botany of the Chinese Academy of Sciences | 501190 |
| *L. craibianus* | The Herbarium of Kunming Institute of Botany of the Chinese Academy of Sciences | 501241 |
| *L. craibianus* | The Herbarium of Kunming Institute of Botany of the Chinese Academy of Sciences | 468793 |
| *L. crassinervius* | The National Herbarium Netherlands | Koorders39442 |
| *L. cryptocarpus* | The Harvard University Herbaria | 8345 |
| *L. cyclophorus* | The National Herbarium Netherlands | Boschproefstation7429 |
| *L. cyclophorus* | The National Herbarium Netherlands | vanSteenis3597 |
| *L. damiaoshanicus* | The Herbarium of Kunming Institute of Botany of the Chinese Academy of Sciences | 501297 |
| *L. dasystachyus* | The National Herbarium Netherlands | Abbe,Smythie&Asah9825 |
| *L. dasystachyus* | The National Herbarium Netherlands | Pasqual2373(207) |
| *L. dasystachyus* | The National Herbarium Netherlands | RidsdalePBU.470 |
| *L. dealbatus* | The Herbarium of Kunming Institute of Botany of the Chinese Academy of Sciences | 671534 |
| *L. dealbatus* | The Herbarium of Kunming Institute of Botany of the Chinese Academy of Sciences | 671568 |
| *L. dealbatus* | The Herbarium of Kunming Institute of Botany of the Chinese Academy of Sciences | 501279 |
| *L. dealbatus* | The Harvard University Herbaria | 3630 |
| *L. dealbatus* | The Harvard University Herbaria | 6505 |
| *L. dealbatus* | The Harvard University Herbaria | 3106 |
| *L. dealbatus* | The Harvard University Herbaria | 62--4 |
| *L. dealbatus* | The Harvard University Herbaria | 62--5 |
| *L. dealbatus* | The Harvard University Herbaria | 7747 |
| *L. dealbatus* | The Harvard University Herbaria | 7682 |
| *L. echinotholus* | The Herbarium of Kunming Institute of Botany of the Chinese Academy of Sciences | 501560 |
| *L. echinotholus* | The Herbarium of Kunming Institute of Botany of the Chinese Academy of Sciences | 501558 |
| *L. edulis* | The Harvard University Herbaria | 25 |
| *L. edulis* | The Harvard University Herbaria | 5805 |
| *L. edulis* | The Harvard University Herbaria | 258 |
| *L. edulis* | The Harvard University Herbaria | 1250 |
| *L. elegans* | The Herbarium of Kunming Institute of Botany of the Chinese Academy of Sciences | 501991 |
| *L. elegans* | The Herbarium of Kunming Institute of Botany of the Chinese Academy of Sciences | 502006 |
| *L. elegans* | The Herbarium of Kunming Institute of Botany of the Chinese Academy of Sciences | 501978 |
| *L. elegans* | The Herbarium of Kunming Institute of Botany of the Chinese Academy of Sciences | 501986 |
| *L. elegans* | The Herbarium of Kunming Institute of Botany of the Chinese Academy of Sciences | 501578 |
| *L. elegans* | The National Herbarium Netherlands | Roelofsen6274 |
| *L. elegans* | The National Herbarium Netherlands | W.F.Winckel286 |
| *L. elegans* | The National Herbarium Netherlands | 1291877 |
| *L. elegans* | The Harvard University Herbaria | 2387 |
| *L. elmerrillii* | The Herbarium of Kunming Institute of Botany of the Chinese Academy of Sciences | 804689 |
| *L. encleisocarpus* | The National Herbarium Netherlands | Grashoff326 |
| *L. encleisocarpus* | The National Herbarium Netherlands | 326 |
| *L. encleisocarpus* | The National Herbarium Netherlands | PaieS.15079 |
| *L. ewyckii* | The National Herbarium Netherlands | AsakakUnyongS.21194 |
| *L. farinulentus* | The Herbarium of Kunming Institute of Botany of the Chinese Academy of Sciences | 502481 |
| *L. fenestratus* | The Herbarium of Kunming Institute of Botany of the Chinese Academy of Sciences | 501610 |
| *L. fenestratus* | The Herbarium of Kunming Institute of Botany of the Chinese Academy of Sciences | 501614 |
| *L. fenestratus* | The Herbarium of Kunming Institute of Botany of the Chinese Academy of Sciences | 501705 |
| *L. fenestratus* | The Herbarium of Kunming Institute of Botany of the Chinese Academy of Sciences | 501753 |
| *L. fenestratus* | The Herbarium of Kunming Institute of Botany of the Chinese Academy of Sciences | 501754 |
| *L. fenestratus* | The Herbarium of Kunming Institute of Botany of the Chinese Academy of Sciences | 786646 |
| *L. fenestratus* | The Herbarium of Kunming Institute of Botany of the Chinese Academy of Sciences | 501718 |
| *L. fenestratus* | The Herbarium of Kunming Institute of Botany of the Chinese Academy of Sciences | 501717 |
| *L. fenestratus* | The Harvard University Herbaria | 4086 |
| *L. fenestratus* | The Harvard University Herbaria | 02-277 |
| *L. ferrugineus* | The National Herbarium Netherlands | Abd.Rahimetal92961 |
| *L. ferrugineus* | The National Herbarium Netherlands | CWL1082 |
| *L. ferrugineus* | The National Herbarium Netherlands | Dyg.Awa&YiiP.C.46720 |
| *L. ferrugineus* | The National Herbarium Netherlands | Aban GibotSAN100016 |
| *L. ferrugineus* | The National Herbarium Netherlands | W.L.Chew1802R |
| *L. ferrugineus* | The National Herbarium Netherlands | JugahakKudi |
| *L. ferrugineus* | The National Herbarium Netherlands | S.Lantoh67719M |
| *L. ferrugineus* | The National Herbarium Netherlands | Tuyuk,Donggop&Amin94395L |
| *L. ferrugineus* | The National Herbarium Netherlands | 57078 |
| *L. fohaiensis* | The Harvard University Herbaria | 1995 1-24-22 |
| *L. fohaiensis* | The Harvard University Herbaria | 79438 |
| *L. fohaiensis* | The Harvard University Herbaria | 74635 |
| *L. formosanus* | The Harvard University Herbaria | 1594 |
| *L. glaber* | The Herbarium of Kunming Institute of Botany of the Chinese Academy of Sciences | 501872 |
| *L. glaber* | The Herbarium of Kunming Institute of Botany of the Chinese Academy of Sciences | 501894 |
| *L. glaber* | The Herbarium of Kunming Institute of Botany of the Chinese Academy of Sciences | 501902 |
| *L. glaber* | The Herbarium of Kunming Institute of Botany of the Chinese Academy of Sciences | 501908 |
| *L. glaber* | The Harvard University Herbaria | 15744 |
| *L. glaber* | The Harvard University Herbaria | 218 |
| *L. glutinosus* | The National Herbarium Netherlands | Elmer20971A |
| *L. gracilis* | The National Herbarium Netherlands | Ashton19449 |
| *L. gracilis* | The National Herbarium Netherlands | Beccari4031 |
| *L. gracilis* | The National Herbarium Netherlands | Clemens26716 |
| *L. gracilis* | The National Herbarium Netherlands | CWL1039 |
| *L. gracilis* | The National Herbarium Netherlands | PaieS.15588 |
| *L. gracilis* | The National Herbarium Netherlands | Villamil216 |
| *L. gracilis* | The National Herbarium Netherlands | Villamil50 |
| *L. gracilis* | The National Herbarium Netherlands | Wood3469 |
| *L. gracilis* | The National Herbarium Netherlands | WoodSan.A4039 |
| *L. hancei* | The Herbarium of Kunming Institute of Botany of the Chinese Academy of Sciences | 502027 |
| *L. hancei* | The Herbarium of Kunming Institute of Botany of the Chinese Academy of Sciences | 502029 |
| *L. hancei* | The Herbarium of Kunming Institute of Botany of the Chinese Academy of Sciences | 695486 |
| *L. hancei* | The Herbarium of Kunming Institute of Botany of the Chinese Academy of Sciences | 502038 |
| *L. hancei* | The Herbarium of Kunming Institute of Botany of the Chinese Academy of Sciences | 691522 |
| *L. hancei* | The Herbarium of Kunming Institute of Botany of the Chinese Academy of Sciences | 691518 |
| *L. hancei* | The Harvard University Herbaria | 10280 |
| *L. hancei* | The Harvard University Herbaria | 11165 |
| *L. hancei* | The Harvard University Herbaria | 10280 |
| *L. hancei* | The Harvard University Herbaria | 11165 |
| *L. hancei* | The Herbarium of Kunming Institute of Botany of the Chinese Academy of Sciences | 502027 |
| *L. hancei* | The Herbarium of Kunming Institute of Botany of the Chinese Academy of Sciences | 502029 |
| *L. hancei* | The Herbarium of Kunming Institute of Botany of the Chinese Academy of Sciences | 695486 |
| *L. hancei* | The Herbarium of Kunming Institute of Botany of the Chinese Academy of Sciences | 502038 |
| *L. hancei* | The Herbarium of Kunming Institute of Botany of the Chinese Academy of Sciences | 691522 |
| *L. hancei* | The Herbarium of Kunming Institute of Botany of the Chinese Academy of Sciences | 691518 |
| *L. handelianus* | The Herbarium of Kunming Institute of Botany of the Chinese Academy of Sciences | 500002 |
| *L. handelianus* | The Herbarium of Kunming Institute of Botany of the Chinese Academy of Sciences | 502305 |
| *L. handelianus* | The Harvard University Herbaria | 34143 |
| *L. handelianus* | The Harvard University Herbaria | 64089 |
| *L. harlandii* | The Harvard University Herbaria | 367 |
| *L. harlandii* | The Harvard University Herbaria | 1026 |
| *L. harlandii* | The Harvard University Herbaria | 732 |
| *L. henryi* | The Harvard University Herbaria | 543 |
| *L. henryi* | The Harvard University Herbaria | 524 |
| *L. himalaicus* | The Herbarium of Kunming Institute of Botany of the Chinese Academy of Sciences | 502422 |
| *L. himalaicus* | The Herbarium of Kunming Institute of Botany of the Chinese Academy of Sciences | 502403 |
| *L. howii* | The Harvard University Herbaria | 26878 |
| *L. hypoglaucus* | The Herbarium of Kunming Institute of Botany of the Chinese Academy of Sciences | 502452 |
| *L. hypoglaucus* | The Herbarium of Kunming Institute of Botany of the Chinese Academy of Sciences | 502441 |
| *L. hypoglaucus* | The Herbarium of Kunming Institute of Botany of the Chinese Academy of Sciences | 502438 |
| *L. indutus* | The National Herbarium Netherlands | 30/10-41 |
| *L. jacobsii* | The National Herbarium Netherlands | 15574 |
| *L. jacobsii* | The National Herbarium Netherlands | IliasPaie15574M |
| *L. javensis* | The National Herbarium Netherlands | Koorders15213 |
| *L. javensis* | The National Herbarium Netherlands | Koorders15338 |
| *L. javensis* | The National Herbarium Netherlands | TorreetHarms1893 |
| *L. kawakamii* | The Harvard University Herbaria | 9653 |
| *L. konishii* | The Harvard University Herbaria | N.H.Li. 271 |
| *L. konishii* | The Harvard University Herbaria | 21326 |
| *L. konishii* | The Harvard University Herbaria | 11174 |
| *L. lampadarius* | The National Herbarium Netherlands | E.C.&L.B.Abbe10002 |
| *L. laoticus* | The Herbarium of Kunming Institute of Botany of the Chinese Academy of Sciences | 502494 |
| *L. lappaceus* | The National Herbarium Netherlands | C.H.Cannon817 |
| *L. lepidocarpus* | The Harvard University Herbaria | 9760 |
| *L. leptogyne* | The National Herbarium Netherlands | Beaman8507 |
| *L. leptogyne* | The National Herbarium Netherlands | CuadraA.1454 |
| *L. leptogyne* | The National Herbarium Netherlands | Elmer21636 |
| *L. leptogyne* | The National Herbarium Netherlands | PaieS.15844 |
| *L. leptogyne* | The National Herbarium Netherlands | WoodSan15437 |
| *L. lindleyanus* | The Herbarium of Kunming Institute of Botany of the Chinese Academy of Sciences | 786766 |
| *L. litseifolius* | The Herbarium of Kunming Institute of Botany of the Chinese Academy of Sciences | 503145 |
| *L. litseifolius* | The Herbarium of Kunming Institute of Botany of the Chinese Academy of Sciences | 503151 |
| *L. litseifolius* | The Harvard University Herbaria | 43505 |
| *L. litseifolius* | The Harvard University Herbaria | 128 |
| *L. litseifolius* | The Harvard University Herbaria | 25865 |
| *L. litseifolius* | The Harvard University Herbaria | 79069 |
| *L. litseifolius* | The Harvard University Herbaria | 27673 |
| *L. longanoides* | The Herbarium of Kunming Institute of Botany of the Chinese Academy of Sciences | 502530 |
| *L. longipedicellatus* | The Herbarium of Kunming Institute of Botany of the Chinese Academy of Sciences | 804684 |
| *L. lucidus* | The National Herbarium Netherlands | Brun5259 |
| *L. luteus* | The National Herbarium Netherlands | Anderson4563 |
| *L. mairei* | The Herbarium of Kunming Institute of Botany of the Chinese Academy of Sciences | 692007 |
| *L. mairei* | The Herbarium of Kunming Institute of Botany of the Chinese Academy of Sciences | 502557 |
| *L. mairei* | The Herbarium of Kunming Institute of Botany of the Chinese Academy of Sciences | 502565 |
| *L. mairei* | The Herbarium of Kunming Institute of Botany of the Chinese Academy of Sciences | 502597 |
| *L. megacarpus* | The National Herbarium Netherlands | L.J.Brass13522 |
| *L. meijeri* | The National Herbarium Netherlands | WrightS.27181 |
| *L. naiadarum* | The Herbarium of Kunming Institute of Botany of the Chinese Academy of Sciences | 502641 |
| *L. naiadarum* | The Herbarium of Kunming Institute of Botany of the Chinese Academy of Sciences | 502654 |
| *L. naiadarum* | The Harvard University Herbaria | 957 |
| *L. naiadarum* | The Harvard University Herbaria | 73995 |
| *L. naiadarum* | The Harvard University Herbaria | 7777 |
| *L. nieuwenhuisii* | The National Herbarium Netherlands | SAN16481 |
| *L. nodosus* | The National Herbarium Netherlands | Clemens29890 |
| *L. oblanceolatus* | The Harvard University Herbaria | 509 |
| *L. oblanceolatus* | The Harvard University Herbaria | 196 |
| *L. obscurus* | The Herbarium of Kunming Institute of Botany of the Chinese Academy of Sciences | 502668 |
| *L. pachylepis* | The Herbarium of Kunming Institute of Botany of the Chinese Academy of Sciences | 772510 |
| *L. pachylepis* | The Herbarium of Kunming Institute of Botany of the Chinese Academy of Sciences | 502696 |
| *L. pachylepis* | The Harvard University Herbaria | 288 |
| *L. pachylepis* | The Harvard University Herbaria | 11500 |
| *L. pachylepis* | The Harvard University Herbaria | 288 |
| *L. pachylepis* | The Harvard University Herbaria | 11500 |
| *L. pachylepis* | The Herbarium of Kunming Institute of Botany of the Chinese Academy of Sciences | 772510 |
| *L. pachylepis* | The Herbarium of Kunming Institute of Botany of the Chinese Academy of Sciences | 502696 |
| *L. pachylepis* | The Herbarium of Kyushu University | v4843 |
| *L. pachyphyllus* | The Herbarium of Kunming Institute of Botany of the Chinese Academy of Sciences | 502679 |
| *L. pallidus* | The National Herbarium Netherlands | Koorders1476B |
| *L. pallidus* | The National Herbarium Netherlands | xxxx1893b |
| *L. petelotii* | The Harvard University Herbaria | 2331 |
| *L. platycarpus* | The National Herbarium Netherlands | Koorders20222B |
| *L. platycarpus* | The National Herbarium Netherlands | Koorders24732B |
| *L. platycarpus* | The National Herbarium Netherlands | 84 |
| *L. polystachyus* | The Herbarium of Kunming Institute of Botany of the Chinese Academy of Sciences | 502791 |
| *L. polystachyus* | The Herbarium of Kunming Institute of Botany of the Chinese Academy of Sciences | 502797 |
| *L. polystachyus* | The Herbarium of Kunming Institute of Botany of the Chinese Academy of Sciences | 502799 |
| *L. polystachyus* | The Herbarium of Kunming Institute of Botany of the Chinese Academy of Sciences | 670308 |
| *L. polystachyus* | The Herbarium of Kunming Institute of Botany of the Chinese Academy of Sciences | 502831 |
| *L. polystachyus* | The Harvard University Herbaria | 1365 |
| *L. polystachyus* | The VNM Herbarium | 12362 |
| *L. pseudokunstleri* | The National Herbarium Netherlands | Cat.#2243 |
| *L. pseudokunstleri* | The National Herbarium Netherlands | S.19936 |
| *L. pseudomoluccus* | The National Herbarium Netherlands | Koorders25864B |
| *L. pseudomoluccus* | The National Herbarium Netherlands | Th.Valeton1912.1 |
| *L. pseudomoluccus* | The National Herbarium Netherlands | v.Gorkum1903.1 |
| *L. pseudovestitus* | The Herbarium of Kunming Institute of Botany of the Chinese Academy of Sciences | 804694 |
| *L. pseudoxizangensis* | The Herbarium of Kunming Institute of Botany of the Chinese Academy of Sciences | 748552 |
| *L. pseudoxizangensis* | The Herbarium of Kunming Institute of Botany of the Chinese Academy of Sciences | 503051 |
| *L. pusillus* | The National Herbarium Netherlands | Burley&Lee267 |
| *L. rhabdostachyus* | The Herbarium of Kunming Institute of Botany of the Chinese Academy of Sciences | 503058 |
| *L. rosthornii* | The Herbarium of Kunming Institute of Botany of the Chinese Academy of Sciences | 503063 |
| *L. silvicolarum* | The Herbarium of Kunming Institute of Botany of the Chinese Academy of Sciences | 503085 |
| *L. silvicolarum* | The Harvard University Herbaria | 73001 |
| *L. silvicolarum* | The Harvard University Herbaria | 15672 |
| *L. silvicolarum* | The Harvard University Herbaria | 1078 |
| *L. skanianus* | The Harvard University Herbaria | 18055 |
| *L. sundaicus* | The National Herbarium Netherlands | Abbe12068 |
| *L. sundaicus* | The National Herbarium Netherlands | Wood17185 |
| *L. taitoensis* | The Herbarium of Kunming Institute of Botany of the Chinese Academy of Sciences | 804234 |
| *L. taitoensis* | The Harvard University Herbaria | 11170 |
| *L. touranensis* | The Herbarium of Kunming Institute of Botany of the Chinese Academy of Sciences | 503187 |
| *L. trachycarpus* | The Harvard University Herbaria | 81134 |
| *L. truncatus* | The Herbarium of Kunming Institute of Botany of the Chinese Academy of Sciences | 503241 |
| *L. truncatus* | The Herbarium of Kunming Institute of Botany of the Chinese Academy of Sciences | 503265 |
| *L. truncatus* | The Herbarium of Kunming Institute of Botany of the Chinese Academy of Sciences | 743185 |
| *L. truncatus* | The Harvard University Herbaria | 333 |
| *L. truncatus* | The Harvard University Herbaria | 75897 |
| *L. truncatus* | The Harvard University Herbaria | 77964 |
| *L. truncatus* | The Harvard University Herbaria | 79110 |
| *L. truncatus* | The Harvard University Herbaria | 77964 |
| *L. truncatus* | The Harvard University Herbaria | 79110 |
| *L. truncatus* | The Herbarium of Kunming Institute of Botany of the Chinese Academy of Sciences | 503241 |
| *L. truncatus* | The Herbarium of Kunming Institute of Botany of the Chinese Academy of Sciences | 503265 |
| *L. truncatus* | The Herbarium of Kunming Institute of Botany of the Chinese Academy of Sciences | 743185 |
| *L. turbinatus* | The National Herbarium Netherlands | J.&M.S.Clemens29884 |
| *L. turbinatus* | The National Herbarium Netherlands | SAN20389 |
| *L. uvariifolius* | The Harvard University Herbaria | 610 |
| *L. uvariifolius* | The Harvard University Herbaria | 25829 |
| *L. uvariifolius* | The Harvard University Herbaria | 25931 |
| *L. variolosus* | The Harvard University Herbaria | 408 |
| *L. variolosus* | The Harvard University Herbaria | 11590 |
| *L. variolosus* | The Harvard University Herbaria | 72006 |
| *L. variolosus* | The Harvard University Herbaria | 6523 |
| *L. variolosus* | The Harvard University Herbaria | 11590 |
| *L. variolosus* | The Harvard University Herbaria | 72006 |
| *L. variolosus* | The Harvard University Herbaria | 6523 |
| *L. vestitus* | The Harvard University Herbaria | 24549 |
