# Supplementary material for: The fruit morphometric variation and fruit type evolution of the stone oaks (Fagaceae, Lithocarpus)
Source: BMC Plant Biol. 2023 Apr 29;23:229. doi: 10.1186/s12870-023-04237-4 (PMC10148511; doi:10.1186/s12870-023-04237-4)
Supplement: Supplementary file 3 — Additional file 3: Figure S3. The Bayesian phylogenetic trees are based on cpDNA + nrITS of 72 species. [file 12870_2023_4237_MOESM3_ESM.docx]

**
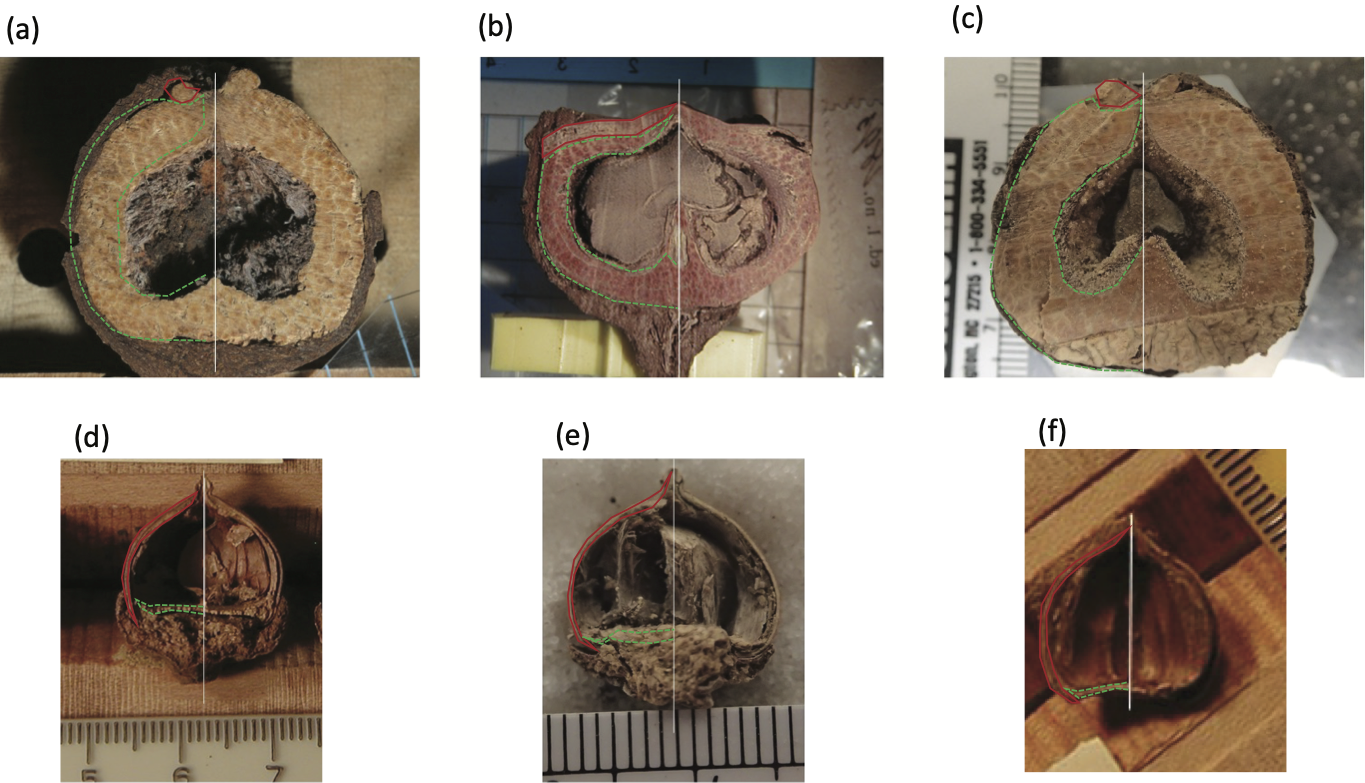
**

**Figure S1.** Fruit morphologies of the two exceptional species with negative allometric slopes. (**a**)-(**c**), ER-type species, *L. javensis*. (**d**)–(**f**), AC-type species, *L. ferrugineus*. Pericarp and receptacle tissues were depicted by solid red lines and dashed green lines on the left side of the longitudinal section respectively.
