# Supplementary material for: The fruit morphometric variation and fruit type evolution of the stone oaks (Fagaceae, Lithocarpus)
Source: BMC Plant Biol. 2023 Apr 29;23:229. doi: 10.1186/s12870-023-04237-4 (PMC10148511; doi:10.1186/s12870-023-04237-4)
Supplement: Supplementary file 4 — Additional file 4: Figure S4. Matching the fruit type to the phylogenetic tree proposed by Yang et. al (2018). Based on the cpDNA + nrITS phylogenetic tree (Fig. 2a) by Yang et al, AC and ER type species were represented by red circles and blue triangles after the species name respectively. The species with unidentified fruit type was not labelled. [file 12870_2023_4237_MOESM4_ESM.docx]

**
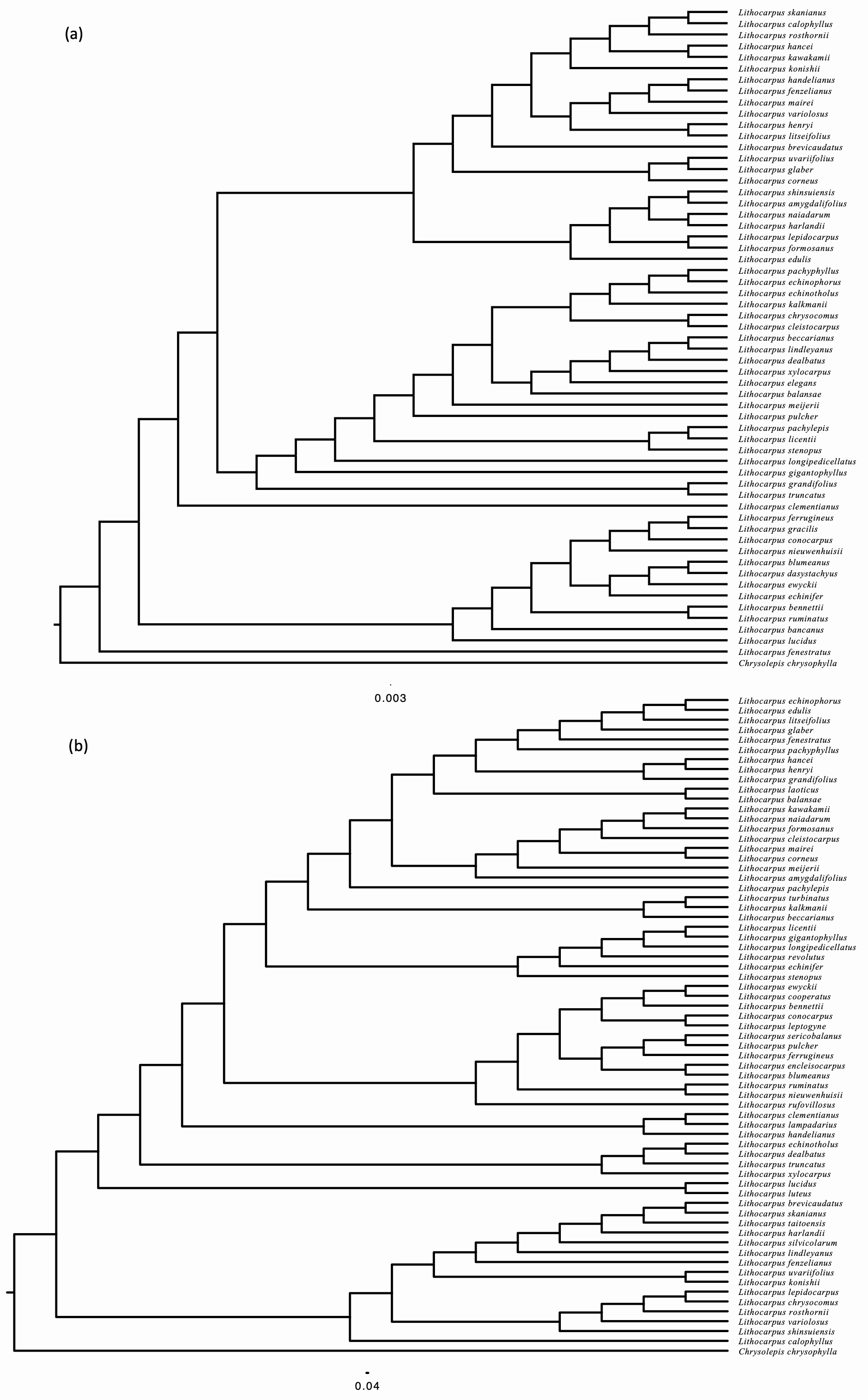
**

**Figure S2.** The ML phylogenetic trees. (**a**) The ML phylogenetic tree based on cpDNA of 58 species. (**b**) The ML phylogenetic tree based on nrITS of 66 species.
