# Supplementary material for: The fruit morphometric variation and fruit type evolution of the stone oaks (Fagaceae, Lithocarpus)
Source: BMC Plant Biol. 2023 Apr 29;23:229. doi: 10.1186/s12870-023-04237-4 (PMC10148511; doi:10.1186/s12870-023-04237-4)
Supplement: Supplementary file 5 — Additional file 5: Figure S5. Ancestral state reconstruction of pericarp and receptacle volume of 72 Lithocarpus species. Estimated ancestral morphometric values are coded by colored branches as explained in the legend within the figure. The species names of AC and ER-type species are colored by black and green respectively. [file 12870_2023_4237_MOESM5_ESM.docx]

**Figure S3.** The Bayesian phylogenetic trees of based on cpDNA + nrITS of 72 species.
