# Supplementary material for: The fruit morphometric variation and fruit type evolution of the stone oaks (Fagaceae, Lithocarpus)
Source: BMC Plant Biol. 2023 Apr 29;23:229. doi: 10.1186/s12870-023-04237-4 (PMC10148511; doi:10.1186/s12870-023-04237-4)
Supplement: Supplementary file 6 — Additional file 6: Figure S6. The four species exhibiting AC-ER intermediate fruit morphology. (a) L. pachylepis. (b) L. lampadarius. (c) L. revolutus. (d) L. pulcher) all represent a similar fruit morphology with unreduced or thickened pericarp (red solid line) and extended and thickened receptacle (green dashed line). [file 12870_2023_4237_MOESM6_ESM.docx]

**Figure S4.** Matching the fruit type to phylogenetic tree proposed by Yang et. al (2018). Based on the cpDNA + nrITS phylogenetic tree (Fig. 2a) by Yang et al, AC and ER type species were represented by red circle and blue triangles after the species name respectively. The species with unidentified fruit type was not labelled.
