# Supplementary material for: The fruit morphometric variation and fruit type evolution of the stone oaks (Fagaceae, Lithocarpus)
Source: BMC Plant Biol. 2023 Apr 29;23:229. doi: 10.1186/s12870-023-04237-4 (PMC10148511; doi:10.1186/s12870-023-04237-4)
Supplement: Supplementary file 7 — Additional file 7: Table S1. The genes fragments and accession number of 72 Lithocarpus species and Chrysolepis chrysophylla applied in the phylogenetic study. [file 12870_2023_4237_MOESM7_ESM.docx]

**Figure S5.** Ancestral state reconstruction of pericarp and receptacle volume of 72 *Lithocarpus* species. Estimated ancestral morphometric values are coded by colored branches as explained in the legend within the figure. The species names of AC and ER-type species are colored by black and green respectively.
