# Supplementary material for: The fruit morphometric variation and fruit type evolution of the stone oaks (Fagaceae, Lithocarpus)
Source: BMC Plant Biol. 2023 Apr 29;23:229. doi: 10.1186/s12870-023-04237-4 (PMC10148511; doi:10.1186/s12870-023-04237-4)
Supplement: Supplementary file 8 — Additional file 8: Table S2. The comparison of species included in our phylogenetic study and study by Yang et al. (2018). [file 12870_2023_4237_MOESM8_ESM.docx]

**
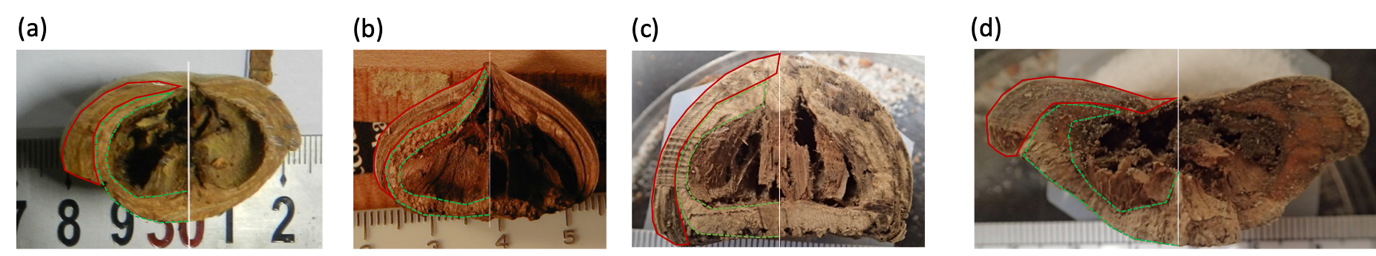
**

**Figure S6.** The four species exhibiting AC-ER intermediate fruit morphology. (**a**) *L. pachylepis*. (**b**) *L. lampadarius*. (**c**) *L. revolutus*. (**d**) *L. pulcher*) all represent a similar fruit morphology with unreduced or thickened pericarp (red solid line) and extended and thickened receptacle (green dashed line).
