# Supplementary material for: The fruit morphometric variation and fruit type evolution of the stone oaks (Fagaceae, Lithocarpus)
Source: BMC Plant Biol. 2023 Apr 29;23:229. doi: 10.1186/s12870-023-04237-4 (PMC10148511; doi:10.1186/s12870-023-04237-4)
Supplement: Supplementary file 9 — Additional file 9: Table S3. The six fruit morphometrics estimated by Pappus-Guldinus Theorem. [file 12870_2023_4237_MOESM9_ESM.docx]

Table S1 The genes fragments and accession number of 72 *Lithocarpus* species and *Chrysolepis chrysophylla* applied in the phylogenetic study

| Fruit type | Species | nrITS | atpB-rbcL | matK | psbA-trnH | rbcL | trnL-trnF |
| --- | --- | --- | --- | --- | --- | --- | --- |
| NA | *Chrysolepis chrysophylla* | AF389087.1 | FJ185062.1 | KF419004.1 | NA | KF418896.1 | KF418961.1 |
| ER | *L. amygdalifolius* | KJ685164.1 | KF992718.1 | KJ687702.1 | KJ686993.1 | KJ688346.1 | NA |
| ER | *L. balansae* | EU195795.1 | NA | NC_026577.1 | NC_026577.1 | NC_026577.1 | NC_026577.1 |
| AC | *L. bancanus* | NA | AB124945.1 | AB125030.1 | NA | AB125013.1 | AB124979.1 |
| ER | *L. beccarianus* | AF389101.1 | AY182835.1 | NA | NA | NA | NA |
| AC | *L. bennettii* | AY040412.1 | AY182838.1 | NA | NA | NA | NA |
| AC | *L. blumeanus* | AF389091.1 | AY182839.1 | NA | NA | NA | NA |
| AC | *L. brevicaudatus* | KJ685165.1 | KF992719.1 | NA | NA | NA | NA |
| AC | *L. calophyllus* | KJ685166.1 | KF992724.1 | NA | NA | NA | NA |
| AC | *L. cantleyanus* | NA | AY182841.1 | NA | NA | NA | NA |
| AC | *L. chrysocomus* | KJ685167.1 | KF992721.1 | NA | NA | NA | NA |
| ER | *L. cleistocarpus* | KJ685168.1 | KF992722.1 | EF057117.1 | NA | NA | EF057148.1 |
| AC | *L. clementianus* | AF389107.1 | AY182844.1 | NA | NA | NA | NA |
| AC | *L. conocarpus* | AY040417.1 | AY182847.1 | NA | NA | NA | NA |
| AC | *L. cooperatus* | AY040406.1 | AY182852.1 | NA | NA | NA | NA |
| ER | *L. corneus* | AY040440.1 | KF992729.1 | NA | NA | NA | NA |
| AC | *L. dasystachyus* | NA | AY182854.1 | NA | NA | NA | NA |
| AC | *L. dealbatus* | AY040430.1 | AY182856.1 | EF057115.1 | NA | NA | EF057150.1 |
| ER | *L. echinifer* | AY040399.1 | AY182858.1 | NA | NA | NA | NA |
| AC | *L. echinophorus* | AY040437.1 | AY182859.1 | EF057116.1 | NA | NA | EF057149.1 |
| AC | *L. echinotholus* | AY040424.1 | AY182860.1 | NA | NA | NA | NA |
| AC | *L. edulis* | AY040439.1 | AY182861.1 | AB060060.1 | LC521808.1 | AB060569.1 | AB063555.1 |
| AC | *L. elegans* | NA | AY182862.1 | NA | NA | NA | NA |
| AC | *L. encleisocarpus* | AY040415.1 | AY182864.1 | NA | NA | NA | NA |
| AC | *L. ewyckii* | AY040413.1 | AY182870.1 | NA | NA | NA | NA |
| AC | *L. fenestratus* | AY040449.1 | AY182872.1 | EF057122.1 | NA | KF418897.1 | EF057161.1 |
| ER | *L. fenzelianus* | KJ685171.1 | KF992731.1 | NA | NA | NA | NA |
| AC | *L. ferrugineus* | AY040414.1 | AY182874.1 | NA | NA | NA | NA |
| AC | *L. formosanus* | KJ685173.1 | KF992732.1 | NA | KJ687406.1 | KJ688821.1 | NA |
| AC | *L. gigantophyllus* | MF770299.1 | NA | LC318538.1 | NA | LC318951.1 | NA |
| AC | *L. glaber* | AY040435.1 | KF992733.1 | AB060059.1 | KP095407.1 | AB060568.1 | AB063554.1 |
| AC | *L. gracilis* | NA | AY182877.1 | KJ708976.1 | NA | NA | NA |
| AC | *L. grandifolius* | AY182879.1 | KR531053.1 | AY040450.1 | KR532875.1 | KR529553.1 | NA |
| AC | *L. hancei* | AY040451.1 | KF992743.1 | LC318970.1 | KP095409.1 | LC318963.1 | NA |
| AC | *L. handelianus* | KJ685185.1 | KF992745.1 | NA | NA | NA | NA |
| AC | *L. harlandii* | KJ685186.1 | KF992746.1 | KJ687755.1 | KJ687093.1 | KJ688473.1 | NA |
| AC | *L. henryi* | EF057110.1 | NA | EF057119.1 | MH059129.1 | AY147097.1 | AY147086.1 |
| AC | *L. jacobsii* | NA | AY182898.1 | NA | NA | NA | NA |
| ER | *L. kalkmanii* | AF389102.1 | AY182902.1 | NA | NA | NA | NA |
| AC | *L. kawakamii* | KJ685190.1 | KF992762.1 | NA | NA | NA | NA |
| AC | *L. konishii* | KJ685192.1 | KF992764.1 | KJ687757.1 | KJ687100.1 | KJ688481.1 | NA |
| ER | *L. lampadarius* | AY040433.1 | AY182904.1 | NA | NA | NA | NA |
| ER | *L. laoticus* | EU195797.1 | NA | NA | NA | NA | NA |
| ER | *L. lepidocarpus* | KJ685193.1 | KF992765.1 | NA | NA | NA | NA |
| AC | *L. leptogyne* | AY040416.1 | NA | NA | NA | NA | NA |
| AC | *L. licentii* | MF770301.1 | NA | LC318540.1 | NA | LC318954.1 | NA |
| AC | *L. lindleyanus* | NA | AY182907.1 | NA | NA | MH113381.1 | NA |
| AC | *L. litseifolius* | EF057112.1 | KF992767.1 | EF057121.1 | KP095412.1 | KP094891.1 | EF057153.1 |
| AC | *L. longipedicellatus* | MF770304.1 | NA | LC318544.1 | NA | LC318958.1 | NA |
| AC | *L. lucidus* | AY040408.1 | AB124948.1 | AB125031.1 | NA | AB125014.1 | AB124980.1 |
| AC | *L. luteus* | AF389096.1 | AY182909.1 | NA | NA | NA | NA |
| AC | *L. mairei* | NA | KF992768.1 | NA | NA | NA | NA |
| AC | *L. meijerii* | NA | AY182911.1 | NA | NA | NA | NA |
| AC | *L. naiadarum* | KJ685195.1 | KF992773.1 | NA | NA | NA | NA |
| AC | *L. nieuwenhuisii* | AY040400.1 | AY182912.1 | NA | NA | NA | NA |
| ER | *L. pachylepis* | AY040441.1 | FJ185065.1 | AY040494.1 | NA | NA | NA |
| AC | *L. pachyphyllus* | AY040447.1 | AY182914.1 | NA | NA | NA | NA |
| ER | *L. pulcher* | AY040423.1 | AY182926.1 | NA | NA | NA | NA |
| ER | *L. revolutus* | AF389098.1 | AY182929.1 | NA | NA | NA | NA |
| AC | *L. rosthornii* | KJ685201.1 | KF992780.1 | NA | NA | NA | NA |
| AC | *L. rufovillosus* | DQ499087.1 | NA | NA | NA | NA | NA |
| ER | *L. ruminatus* | AF389097.1 | AY182937.1 | KU509035.1 | KU509019.1 | NA | KU509017.1 |
| AC | *L. sericobalanus* | AY040419.1 | AY182939.1 | NA | NA | NA | NA |
| AC | *L. shinsuiensis* | KJ685202.1 | KF992782.1 | NA | NA | NA | NA |
| AC | *L. silvicolarum* | KJ685204.1 | KF992783.1 | NA | NA | NA | NA |
| AC | *L. skanianus* | KJ685206.1 | KF992784.1 | NA | NA | NA | NA |
| AC | *L. stenopus* | MF770300.1 | NA | LC318539.1 | NA | LC318952.1 | NA |
| AC | *L. taitoensis* | KJ685200.1 | KF992778.1 | NA | NA | NA | NA |
| ER | *L. truncatus* | AY040428.1 | AY182944.1 | KR531054.1 | KR532840.1 | KR529557.1 | NA |
| ER | *L. turbinatus* | AY040398.1 | AY182948.1 | NA | NA | NA | NA |
| ER | *L. uvariifolius* | KJ685211.1 | KF992792.1 | NA | NA | KP094607.1 | NA |
| ER | *L. variolosus* | NA | AY182949.1 | KR531056.1 | KR532871.1 | KR529559.1 | NA |
| ER | *L. xylocarpus* | AY040426.1 | AY182950.1 | AY040493.1 | KP968676.1 | NA | NA |

NA stand for the gene fragments are not available.
