# Supplementary material for: The fruit morphometric variation and fruit type evolution of the stone oaks (Fagaceae, Lithocarpus)
Source: BMC Plant Biol. 2023 Apr 29;23:229. doi: 10.1186/s12870-023-04237-4 (PMC10148511; doi:10.1186/s12870-023-04237-4)
Supplement: Supplementary file 10 — Additional file 10: Table S4. The distribution of 72 species from the plants of the world online (https://powo.science.kew.org/). [file 12870_2023_4237_MOESM10_ESM.docx]

Table S2 The comparison of species included in our phylogenetic study and study by Yang et al. (2018)

| Yang et al. 2018 cpDNA+nrITS 64species | cpDNA+nrITS 72 species | cpDNA 58 species | nrITS 66 species |
| --- | --- | --- | --- |
| *L. amygdalifolius* | *L. amygdalifolius* | *L. amygdalifolius* | *L. amygdalifolius* |
| NA | *L. balansae* | *L. balansae* | *L. balansae* |
| NA | *L. bancanus* | *L. bancanus* | NA |
| *L. beccarianus* | *L. beccarianus* | *L. beccarianus* | *L. beccarianus* |
| *L. bennettii* | *L. bennettii* | *L. bennettii* | *L. bennettii* |
| *L. blumeanus* | *L. blumeanus* | *L. blumeanus* | *L. blumeanus* |
| *L. brevicaudatus* | *L. brevicaudatus* | *L. brevicaudatus* | *L. brevicaudatus* |
| *L. bullatus* | NA | NA | NA |
| *L. calophyllus* | *L. calophyllus* | *L. calophyllus* | *L. calophyllus* |
| NA | *L. cantleyanus* | Identical to *L. bennettii* | NA |
| *L. chrysocomus* | *L. chrysocomus* | *L. chrysocomus* | *L. chrysocomus* |
| *L. cleistocarpus* | *L. cleistocarpus* | *L. cleistocarpus* | *L. cleistocarpus* |
| *L. clementianus* | *L. clementianus* | *L. clementianus* | *L. clementianus* |
| *L. conocarpus* | *L. conocarpus* | *L. conocarpus* | *L. conocarpus* |
| *L. cooperatus* | *L. cooperatus* | Identical to *L. bennettii* | *L. cooperatus* |
| *L. cornea* | *L. corneus* | *L. corneus* | *L. corneus* |
| NA | *L. dasystachyus* | *L. dasystachyus* | NA |
| *L. dealbatus* | *L. dealbatus* | *L. dealbatus* | *L. dealbatus* |
| *L. dodoniifolia* | NA | NA | NA |
| L. echinifer | *L. echinifer* | *L. echinifer* | *L. echinifer* |
| *L. echinophorus* | *L. echinophorus* | *L. echinophorus* | *L. echinophorus* |
| *L. echinotholus* | *L. echinotholus* | *L. echinotholus* | *L. echinotholus* |
| *L. edulis* | *L. edulis* | *L. edulis* | *L. edulis* |
| NA | *L. elegans* | *L. elegans* | NA |
| *L. encleisocarpus* | *L. encleisocarpus* | Identical to *L. beccarianus* | *L. encleisocarpus* |
| *L. ewyckii* | *L. ewyckii* | *L. ewyckii* | *L. ewyckii* |
| *L. fenestratus* | *L. fenestratus* | *L. fenestratus* | *L. fenestratus* |
| *L. fenzelianus* | *L. fenzelianus* | *L. fenzelianus* | *L. fenzelianus* |
| *L. ferrugineus* | *L. ferrugineus* | *L. ferrugineus* | *L. ferrugineus* |
| *L. formosana* | *L. formosanus* | *L. formosanus* | *L. formosanus* |
| NA | *L. gigantophyllus* | *L. gigantophyllus* | *L. gigantophyllus* |
| L. glaber | *L. glaber* | *L. glaber* | *L. glaber* |
| NA | *L. gracilis* | *L. gracilis* | NA |
| *L. grandifolius* | *L. grandifolius* | *L. grandifolius* | *L. grandifolius* |
| *L. hancei* | *L. hancei* | *L. hancei* | *L. hancei* |
| *L. handelianus* | *L. handelianus* | *L. handelianus* | *L. handelianus* |
| *L. harlandii* | *L. harlandii* | *L. harlandii* | *L. harlandii* |
| *L. hatusimae* | NA | NA | NA |
| *L. havilandii* | NA | NA | NA |
| NA | *L. henryi* | *L. henryi* | *L. henryi* |
| NA | *L. jacobsii* | Identical to *L. beccarianus* | NA |
| *L. kalkmanii* | *L. kalkmanii* | *L. kalkmanii* | *L. kalkmanii* |
| *L. kawakamii* | *L. kawakamii* | *L. kawakamii* | *L. kawakamii* |
| *L. keningauensis* | NA | NA | NA |
| *L. konishii* | *L. konishii* | *L. konishii* | *L. konishii* |
| *L. lampadarius* | *L. lampadarius* | Identical to *L. beccarianus* | *L. lampadarius* |
| NA | *L. laoticus* | NA | *L. laoticus* |
| *L. lepidocarpus* | *L. lepidocarpus* | *L. lepidocarpus* | *L. lepidocarpus* |
| NA | *L. leptogyne* | NA | *L. leptogyne* |
| NA | *L. licentii* | *L. licentii* | *L. licentii* |
| NA | *L. lindleyanus* | *L. lindleyanus* | *L. lindleyanus* |
| *L. litseifolius* | *L. litseifolius* | *L. litseifolius* | *L. litseifolius* |
| NA | *L. longipedicellatus** | *L. longipedicellatus** | *L. longipedicellatus** |
| *L. lucidus* | *L. lucidus* | *L. lucidus* | *L. lucidus* |
| *L. luteus* | *L. luteus* | Identical to *L. beccarianus* | *L. luteus* |
| *L. mairei* | *L. mairei* | *L. mairei* | *L. mairei* |
| NA | *L. meijeri* | *L. meijeri* | *L. meijeri* |
| *L. naiadarum* | *L. naiadarum* | *L. naiadarum* | *L. naiadarum* |
| *L. nantoensis* | NA | NA | NA |
| *L. nieuwenhuisii* | *L. nieuwenhuisii* | *L. nieuwenhuisii* | *L. nieuwenhuisii* |
| *L. oleaefolius* | NA | NA | NA |
| *L. pachylepis* | *L. pachylepis* | *L. pachylepis* | *L. pachylepis* |
| *L. pachyphyllus* | *L. pachyphyllus* | *L. pachyphyllus* | *L. pachyphyllus* |
| *L. palungensis* | NA | NA | NA |
| *L. paniculatus* | NA | NA | NA |
| *L. papillifer* | NA | NA | NA |
| *L. pulcher* | *L. pulcher* | *L. pulcher* | *L. pulcher* |
| NA | *L. revolutus* | Identical to *L. beccarianus* | *L. revolutus* |
| *L. rosthornis* | *L. rosthornii* | *L. rosthornii* | *L. rosthornii* |
| *L. rotundatus* | NA | NA | NA |
| NA | *L. rufovillosus* | NA | *L. rufovillosus* |
| *L. ruminatus* | *L. rumitus* | *L. rumitus* | *L. rumitus* |
| *L. sericobalanus* | *L. sericobalanos* | Identical to *L. beccarianus* | *L. sericobalanos* |
| *L. shinsuiensis* | *L. shinsuiensis* | *L. shinsuiensis* | *L. shinsuiensis* |
| *L. sivicolarum* | *L. silvicolarum* | Identical to *L. brevicaudatus* | *L. silvicolarum* |
| *L. skanianus* | *L. skanianus* | *L. skanianus* | *L. skanianus* |
| NA | *L. stenopus* | *L. stenopus* | *L. stenopus* |
| *L. taitoensis* | *L. taitoensis* | Identical to *L. brevicaudatus* | *L. taitoensis* |
| *L. truncatus* | *L. truncatus* | *L. truncatus* | *L. truncatus* |
| *L. turbinatus* | *L. turbinatus* | Identical to *L. beccarianus* | *L. turbinatus* |
| *L. uvarifolius* | *L. uvariifolius* | *L. uvariifolius* | *L. uvariifolius* |
| NA | *L. variolosus* | *L. variolosus* | *L. variolosus* |
| *L. xylocarpus* | *L. xylocarpus* | *L. xylocarpus* | *L. xylocarpus* |

The identical sequences were removed for the phylogenetic analysis.
