# Supplementary material for: The fruit morphometric variation and fruit type evolution of the stone oaks (Fagaceae, Lithocarpus)
Source: BMC Plant Biol. 2023 Apr 29;23:229. doi: 10.1186/s12870-023-04237-4 (PMC10148511; doi:10.1186/s12870-023-04237-4)
Supplement: Supplementary file 11 — Additional file 11: Table S5. The deposition numbers of the dissected fruit samples from the herbarium specimens. [file 12870_2023_4237_MOESM11_ESM.docx]

Table S3 The six fruit morphometrics estimated by Pappus-Guldinus Theorem

| Fruit type | Species | Sample | S_p_ | S_r_ | S_s_ | V_p_ | V_r_ | V_s_ |
| --- | --- | --- | --- | --- | --- | --- | --- | --- |
|  |  | number | (cm^2^) | (cm^2^) | (cm^2^) | (cm^3^) | (cm^3^) | (cm^3^) |
| AC | *L. bancanus* | 9 | 8.08 | 1.96 | 10.13 | 0.76 | 0.14 | 2.37 |
| AC | *L. bennettii* | 12 | 6.57 | 1.43 | 7.11 | 0.41 | 0.13 | 1.45 |
| AC | *L. blumeanus* | 14 | 6.07 | 1.98 | 7.87 | 0.56 | 0.15 | 1.40 |
| AC | *L. brevicaudatus* | 5 | 10.69 | 2.75 | 12.81 | 2.24 | 0.39 | 4.16 |
| AC | *L. calophyllus* | 1 | 6.43 | 2.25 | 6.88 | 1.10 | 0.15 | 1.81 |
| AC | *L. cantleyanus* | 3 | 5.39 | 0.64 | 6.13 | 0.31 | 0.04 | 1.17 |
| AC | *L. chrysocomus* | 1 | 4.03 | 2.20 | 5.80 | 0.32 | 0.17 | 1.14 |
| AC | *L. clementianus* | 4 | 8.17 | 3.12 | 9.56 | 1.04 | 0.33 | 2.06 |
| AC | *L. conocarpus* | 11 | 4.79 | 0.88 | 5.49 | 0.39 | 0.06 | 1.03 |
| AC | *L. cooperatus* | 11 | 7.01 | 2.07 | 8.69 | 0.66 | 0.17 | 1.80 |
| AC | *L. dasystachyus* | 3 | 3.96 | 0.36 | 4.15 | 0.23 | 0.02 | 0.67 |
| AC | *L. dealbatus* | 216 | 3.54 | 2.47 | 5.47 | 0.31 | 0.20 | 1.10 |
| AC | *L. echinophorus* | 11 | 10.07 | 3.98 | 12.14 | 1.58 | 0.46 | 3.07 |
| AC | *L. echinotholus* | 5 | 8.37 | 2.49 | 10.06 | 0.79 | 0.12 | 2.42 |
| AC | *L. edulis* | 8 | 7.06 | 1.83 | 7.64 | 0.60 | 0.15 | 1.83 |
| AC | *L. elegans* | 68 | 10.65 | 3.73 | 12.47 | 2.30 | 0.51 | 3.37 |
| AC | *L. encleisocarpus* | 25 | 12.36 | 4.13 | 14.83 | 2.23 | 0.64 | 4.77 |
| AC | *L. ewyckii* | 12 | 7.79 | 1.57 | 8.80 | 0.70 | 0.19 | 2.12 |
| AC | *L. fenestratus* | 153 | 4.43 | 0.93 | 5.27 | 0.40 | 0.06 | 0.98 |
| AC | *L. ferrugineus* | 17 | 5.49 | 1.16 | 6.27 | 0.35 | 0.14 | 1.18 |
| AC | *L. formosanus* | 2 | 7.49 | 1.54 | 6.86 | 1.04 | 0.15 | 1.51 |
| AC | *L. gigantophyllus* | 1 | 7.66 | 3.37 | 8.26 | 1.05 | 0.52 | 1.63 |
| AC | *L. glaber* | 31 | 6.45 | 0.94 | 6.79 | 0.56 | 0.08 | 1.45 |
| AC | *L. gracilis* | 22 | 7.41 | 2.20 | 9.32 | 0.69 | 0.15 | 2.27 |
| AC | *L. grandifolius* | 53 | 7.76 | 3.44 | 9.68 | 1.29 | 0.29 | 2.39 |
| AC | *L. hancei* | 242 | 8.25 | 2.00 | 8.96 | 0.88 | 0.15 | 2.27 |
| AC | *L. handelianus* | 12 | 9.18 | 3.06 | 12.01 | 1.37 | 0.30 | 2.82 |
| AC | *L. harlandii* | 14 | 9.45 | 1.52 | 9.57 | 1.46 | 0.19 | 2.69 |
| AC | *L. henryi* | 11 | 8.13 | 1.87 | 8.48 | 0.85 | 0.13 | 2.05 |
| AC | *L. jacobsii* | 4 | 15.57 | 3.80 | 14.35 | 1.04 | 0.36 | 3.68 |
| AC | *L. kawakamii* | 9 | 12.90 | 5.21 | 13.67 | 2.49 | 0.79 | 4.54 |
| AC | *L. konishii* | 19 | 7.92 | 5.30 | 4.97 | 1.62 | 1.64 | 0.98 |
| AC | *L. leptogyne* | 16 | 4.49 | 1.41 | 5.46 | 0.30 | 0.09 | 0.86 |
| AC | *L. licentii* | 8 | 9.55 | 3.96 | 10.71 | 1.77 | 0.55 | 2.30 |
| AC | *L. lindleyanus* | 5 | 4.97 | 0.87 | 4.94 | 0.62 | 0.06 | 0.91 |
| AC | *L. litseifolius* | 24 | 6.39 | 2.35 | 6.93 | 0.83 | 0.25 | 1.44 |
| AC | *L. longipedicellatus* | 18 | 5.06 | 1.82 | 5.87 | 0.54 | 0.13 | 1.03 |
| AC | *L. lucidus* | 13 | 8.21 | 6.05 | 11.43 | 1.87 | 1.26 | 2.68 |
| AC | *L. luteus* | 1 | 6.79 | 1.88 | 6.74 | 0.82 | 0.36 | 1.19 |
| AC | *L. mairei* | 14 | 4.19 | 0.66 | 4.78 | 0.29 | 0.04 | 0.94 |
| AC | *L. meijerii* | 5 | 6.77 | 0.97 | 9.22 | 0.62 | 0.10 | 1.48 |
| AC | *L. naiadarum* | 16 | 6.32 | 1.00 | 7.17 | 0.53 | 0.09 | 1.63 |
| AC | *L. nieuwenhuisii* | 1 | 9.88 | 0.32 | 9.13 | 1.50 | 0.02 | 2.46 |
| AC | *L. pachyphyllus* | 53 | 7.03 | 3.77 | 8.80 | 1.27 | 0.36 | 2.26 |
| AC | *L. rosthornii* | 3 | 5.49 | 0.69 | 6.55 | 0.29 | 0.03 | 1.25 |
| AC | *L. rufovillosus* | 18 | 13.37 | 3.97 | 15.56 | 2.40 | 0.66 | 4.97 |
| AC | *L. sericobalanus* | 4 | 10.59 | 5.48 | 13.52 | 2.73 | 1.89 | 3.33 |
| AC | *L. shinsuiensis* | 4 | 9.17 | 2.73 | 10.65 | 1.03 | 0.18 | 2.59 |
| AC | *L. silvicolarum* | 25 | 10.43 | 3.91 | 13.22 | 2.10 | 0.39 | 3.62 |
| AC | *L. skanianus* | 2 | 6.94 | 1.59 | 8.24 | 0.85 | 0.14 | 1.45 |
| AC | *L. stenopus* | 6 | 5.06 | 1.82 | 5.56 | 0.57 | 0.18 | 0.95 |
| AC | *L. taitoensis* | 7 | 6.37 | 1.74 | 6.80 | 0.66 | 0.13 | 1.38 |
| ER | *L. amygdalifolius* | 16 | 6.58 | 9.89 | 11.65 | 1.06 | 2.14 | 3.17 |
| ER | *L. balansae* | 1 | 3.34 | 8.78 | 11.08 | 0.32 | 1.47 | 2.41 |
| ER | *L. beccarianus* | 5 | 2.41 | 9.52 | 9.86 | 0.26 | 4.07 | 2.18 |
| ER | *L. cleistocarpus* | 11 | 3.16 | 6.08 | 7.12 | 0.27 | 0.54 | 1.61 |
| ER | *L. corneus* | 25 | 10.33 | 18.16 | 17.53 | 3.78 | 13.31 | 6.27 |
| ER | *L. echinifer* | 9 | 8.26 | 13.91 | 18.04 | 1.73 | 2.73 | 5.57 |
| ER | *L. fenzelianus* | 1 | 2.59 | 7.24 | 7.27 | 0.19 | 0.83 | 1.49 |
| ER | *L. kalkmanii* | 2 | 5.88 | 22.53 | 22.60 | 4.99 | 14.94 | 6.69 |
| ER | *L. lampadarius* | 1 | 15.32 | 16.54 | 15.05 | 3.21 | 4.67 | 4.32 |
| ER | *L. laoticus* | 2 | 1.94 | 9.67 | 10.03 | 0.30 | 1.66 | 3.42 |
| ER | *L. lepidocarpus* | 8 | 2.50 | 13.92 | 14.24 | 0.53 | 3.72 | 5.53 |
| ER | *L. pachylepis* | 74 | 19.61 | 22.27 | 21.49 | 6.20 | 8.59 | 7.54 |
| ER | *L. pulcher* | 2 | 27.11 | 31.21 | 26.42 | 10.99 | 18.15 | 9.41 |
| ER | *L. revolutus* | 4 | 37.58 | 42.90 | 42.12 | 13.55 | 17.11 | 16.19 |
| ER | *L. ruminatus* | 1 | 7.04 | 11.77 | 10.43 | 1.05 | 4.41 | 4.09 |
| ER | *L. truncatus* | 60 | 1.59 | 4.70 | 5.25 | 0.15 | 0.52 | 1.07 |
| ER | *L. turbinatus* | 3 | 8.38 | 24.64 | 22.24 | 5.74 | 28.40 | 12.94 |
| ER | *L. uvariifolius* | 5 | 10.46 | 11.24 | 12.13 | 2.98 | 4.81 | 3.67 |
| ER | *L. variolosus* | 27 | 5.31 | 6.45 | 9.17 | 0.75 | 0.72 | 2.24 |
| ER | *L. xylocarpus* | 162 | 5.02 | 13.74 | 14.26 | 0.69 | 2.39 | 4.69 |

S_s_, S_p_, S_r_, V_p_, V_r_ and V_s_ stand for the surface area of the seed space, the coverage by pericarp, the coverage by receptacle, volume of pericarp, receptacle and seed space respectively.
